# The concomitant administration of systemic amoxicillin and metronidazole compared to scaling and root planing alone in treating periodontitis: =a systematic review=

**DOI:** 10.1186/s12903-015-0123-6

**Published:** 2016-02-29

**Authors:** Dina Zandbergen, Dagmar Else Slot, Richard Niederman, Fridus A. Van der Weijden

**Affiliations:** Academic Centre for Dentistry Amsterdam (ACTA), University of Amsterdam and VU University, Gustav Mahlerlaan 3004, 1081 LA Amsterdam, The Netherlands; College of Dentistry, New York University, 345 E. 24th Street, New York, NY 10010 USA; Clinic for Periodontology, Livingstonelaan 466, 3526 JB Utrecht, The Netherlands

**Keywords:** Periodontal therapy, Scaling and root planning, Systemic antibiotics, Amoxicillin, Metronidazole, Periodontitis, Antimicrobial drug, Non-surgical periodontal therapy, Systematic review, Evidence based dentistry

## Abstract

**Background:**

The treatment of periodontitis begins with a non-surgical phase that includes scaling and root planing(SRP) and on occasion the use of systemic antibiotics. The goal was to systematically evaluate in systemic healthy adults the effect of the concomitant administration of amoxicillin (amx) and metronidazole (met) adjunctive to SRP compared to SRP alone.

**Methods:**

The PubMed-MEDLINE, Cochrane-CENTRAL and EMBASE databases were searched up to November 2014 to identify appropriate studies. Probing Pocket Depth (PD), Clinical Attachment Level (CAL), Bleeding on Pocket Probing(BOP) and Plaque Indices(PI) were selected as outcome variables. Based on the extracted data a meta-analysis was conducted.

**Results:**

A total of 526 unique articles were found, 20 studies met the eligibility criteria. A meta-analysis showed that SRP + amx + met provided significantly better effects overall and more pronounced PD reduction in periodontal pockets initially measuring ≥6 mm (DiffM:-0.86 mm, *p* < 0.00001) and gain in CAL(DiffM:+0.75 mm, *p* = 0.0001). The meta-analysis for the secondary inflammatory parameter BOP showed that SRP + amx + met provided full mouth significantly greater reduction in BOP than SRP alone (DiffM:-6.98 %, *p* = 0.0001).

**Conclusion:**

Adjunctive systemic amoxicillin and metronidazole medication to SRP significantly improved the clinical outcomes with respect to mean PD, CAL and BOP compared to SRP alone. There is moderate to strong evidence in support of the recommendation that adjunctive amx + met therapy to SRP significantly improves the clinical outcomes, with respect to mean PD and CAL compared to SRP alone especially in initially deep (≥6 mm) pockets. No major side effects associated with the intake of amx + met were reported. This treatment regimen is an efficacious, minimally invasive, practical and inexpensive approach for periodontitis therapy. The key components are mechanical tooth and pocket debridement, supportive treatment of the disease with systemic antibiotics and attention to proper self-care.

**Electronic supplementary material:**

The online version of this article (doi:10.1186/s12903-015-0123-6) contains supplementary material, which is available to authorized users.

## Background

Periodontitis is a bacterial infection resulting in a secondary inflammatory response. This inflammatory response negatively affects the surrounding periodontal ligament and alveolar bone. If untreated the resulting loss of attachment structures can ultimately lead to tooth loss [1]. In this context periodontitis can be seen as an alteration from a eubiotic human microbiome and inflammatory response to dysbiosis. Further this dysbiosis can have adverse effects on systemic health [1].

The microbiota responsible for periodontal diseases are complex [2]. Bacterial species adhere to the tooth surface and are organized in a complex structure, the dental plaque biofilm [3]. Mechanical treatment of of periodontal disease is aimed at reducing/eliminating this subgingival plaque and calculus, and/or surgically reducing the periodontal pocket [4]. This reduces the microbial load, short term, but no effect on the ratios of healthy to disease related micobiome [5]. The attempt to suppress the subgingival microbiota, as much as possible, favours repair and regeneration of the periodontium [6]. In numerous short- and long term clinical trials non-surgical periodontal therapy, combined with effective supragingival plaque control, has been shown to be effective [7, 8]. However scaling and root planning(SRP) does not always lead to the microbiological changes necessary for maintaining the long-term stability of the clinical benefits achieved initially [9, 10].

Adjunctive systemic antimicrobials have the potential to affect periodontal pathogens via gingival crevicular fluid at subgingival areas insufficiently affected by mechanical instrumentation [11]. Preferably, a new microbial community must be established in the subgingival biofilm, with higher levels and proportions of microorganisms compatible with periodontal health [12]. Adjunctive antimicrobial therapy may enhance the treatment effect [13]. The combination of metronidazole and amoxicillin(amx + met), as first introduced in periodontology by van Winkelhoff et al. [14], has attracted considerable research and clinical interest [15]. This combination of systemic antibiotics and a strict control of supragingival plaque during the active phase of therapy has shown promising results in the treatment of chronic periodontitis [12]. Combining amx + met results in a synergistic bactericidal effect that in turn reduces the time and dosage level required to obtain optimal effect, and ultimately minimizes the toxicity of both drugs. It is also known that hydroxymetabolite of metronidazole, which is produced in the human liver. It has been suggested that the combination of metronidazole and its hydroxymetabolite acts synergistically [16].

Recently a systematic-review(SR) was published [17] which included 28 clinical trials estimating in a meta-analysis what may be expected as the treatment effect from baseline to end-trial following SRP + amx + met therapy. The present meta-analysis considering clinical parameters of periodontitis was initiated to review in comparison to SRP alone, the complementary effect of SRP + amx + met in patients with periodontitis. Additionally the occurrence of adverse events was evaluated.

## Methods

This SR was conducted in accordance with the guidelines of Transparent Reporting of Systematic Reviews and Meta-analyses(PRISMA-statement) [18]. The protocol detailing the review method was developed “a priori” following initial discussion between members of the research team.

### Focused question

In patients with periodontitis what is the effect of concomitant systemic administration of amoxicillin and metronidazole as an adjunct to SRP compared to SRP alone with respect to mean treatment outcome(end scores versus baseline) in terms of pocket depth(PD), clinical attachment level(CAL), bleeding on probing(BOP), and plaque indices(PI)? Furthermore is the administration of antibiotics associated with side effects?

### Search strategy

Three internet sources were used to search for studies conducted in the period up to and including November 2014 that satisfied the study purpose. These databases included MEDLINE-PubMed, EMBASE and Cochrane-CENTRAL. The search was designed to include any appropriate published study that evaluated amx + met in the treatment of periodontitis (Table 1). In addition the Journal of Dental Research, the Journal of Periodontology, the Journal of Clinical Periodontology, the Journal of Periodontal Research, the European Journal of Oral Sciences were searched for ‘early view’ non-indexed studies.

**Table 1:** Search terms used for PubMed-MEDLINE, Cochrane-CENTRAL and EMBASE. The search strategy was customized according to the database being searched

*Intervention:*
{(<Amoxicillin AND Metronidazole [MeSH] > OR < Amoxicillin AND Metronidazole [textwords]>)
AND
*Outcome:*
(Periodontal Pocket OR Gingival Pocket OR Periodontal Diseases [MeSH] OR Periodontitis OR periodontal disease OR periodontal diseas* OR pocket depth OR pocket-depth OR periodontal attachment loss OR periodontal pocket OR gingival pocket OR gingival pockets OR periodontal pocket OR periodontal pockets OR clinical attachment loss OR pockets OR probing depth OR probing-depth OR probing-pocket-depth OR probing pocket depth OR papillary bleeding index OR sulcus bleeding OR bleeding on probing OR gingival bleeding OR bleeding on probing OR papillary bleeding index OR bleeding index OR gingival index OR gingival inflammation OR gingival diseases* OR gingivitis [textwords])}

### Eligibility criteria

The following eligibility criteria were imposed for inclusion in the SR:

- Randomized controlled clinical trials(RCT’s) or controlled clinical trials(CCT’s)
- Participants: In good general health (no systemic disorders or pregnancy)
- Humans with untreated periodontitis (not treated for ≥6 months)
- Intervention: SRP + amx + met compared to SRP alone.
- Clinical parameters of interest: PD and CAL alterations as primary outcome parameters. BOP and PI changes as secondary outcome parameters.
- Minimum follow up ≥ 2 months.
- Mean pre- and post-treatment outcomes as well as incremental data.

### Selection strategy

The papers were independently screened by title and abstract by two reviewers(DZ&GAW). Papers written in English and Dutch were accepted. If the search keywords and relevant eligibility criteria were present in the title and/or the abstract the paper was selected for full text reading. Papers without abstracts but with titles suggesting that they were related to the objectives of this review were also selected for full text screening. Full-text papers were read in detail by two reviewers(DZ&GAW) and papers that fulfilled all of the selection criteria were processed for data extraction. The reference lists of all selected studies were hand searched for additional relevant articles and available systematic reviews(SR). Disagreements between the two reviewers were resolved by discussion, if persisted the judgment of a third reviewer(DES) was decisive.

### Assessment of heterogeneity

Factors used to evaluate the heterogeneity of the characteristics of the different studies were as follows: study design, participants, interventions, and adverse events.

### Quality assessment

Two reviewers(DES&DZ) scored the methodological qualities of the included studies. The methodological study quality was assessed according to the RCT-checklist of the Dutch Cochrane Center [19] and according to additional quality criteria that were obtained from the CONSORT-statement [20], Moher et al. [21, 22], Needleman et al. [23], the Jadad-scale [24] and the Delphi-List [25]. Criteria were designated for each domain of the internal validity, external validity and statistical methods.

### Data extraction and analysis

Mean and standard deviations (SD), were extracted using data extraction forms (DZ&DES). Any disagreement was discussed, if persisted the judgment of a third reviewer (GAW) was decisive. Some of the papers provided standard errors (SE) of the mean. For which the SD was calculated based on the following formula (SE = SD/√N). When intermediate assessments were performed the longest evaluation period was considered. For those articles that provided insufficient data the first or corresponding author was contacted for additional data. To warrant a precise estimate any data approximation in figures was avoided.

Primary parameters were PD and CAL. BOP and PI were assessed as secondary parameters. Where possible a quantitative analysis and subsequent meta-analysis (MA) was performed summarizing between group outcomes at the baseline and end of trial assessments in a difference of means (DiffM) with the associated 95 % confidence interval. [Review Manager (RevMan, Version 5.1; The Nordic Cochrane Centre, The Cochrane Collaboration, Copenhagen, Denmark, 2011]. A “random or fixed effects” model was used where appropriate. If there were ≤ four studies a “fixed-effect” analysis was performed [26]. Heterogeneity was tested by chi-square-test and the I^2^-statistic. The formal testing for publication bias as proposed by Egger et al. [27] was performed when ≥10 studies were included in the MA (Higgins & Green [26]). In addition the collective data of all individual included studies was summarized and presented in a descriptive manner.

### Grading the body of evidence

The Grading of Recommendations Assessment, Development and Evaluation (GRADE) system as proposed by the GRADE-working group [28] was used to appraise the evidence emerging from this review. Two reviewers (GAW&DES) rated the quality of the evidence and the strength of the recommendations. Any disagreement between the two reviewers was resolved after additional discussion.

## Results

### Search and selection

The search identified 526 unique papers (Additional file 1: S1). The screening of titles and abstracts initially resulted in 64 full-text articles of which 33 papers, after full text reading, were excluded for failing the eligibility criteria (Additional file 1: S2). Subsequently, 31 studies were selected for inclusion in this review. Some studies described the same experiment and provided identical data. After combining these studies, 20 clinical studies remained.

### Study characteristics and heterogeneity

Detailed information regarding the study outline of the selected papers is presented in Additional file 1: S3. In general a considerable heterogeneity in the design, characteristics of participants and their smoking status, intervention regimens and adverse events was present (see Additional file 1: S4-7).

### Side effects

The prevalence of adverse events in patients treated with systemic antimicrobials varied greatly. Most adverse events reported were gastrointestinal. The complaints included nausea, vomiting, headache and metallic taste (for details see Additional file 1: S8)

### Quality assessment

Detailed information regarding the results of the quality assessment of the selected studies is provided in Additional file 1: S10. Formal testing for publication bias was limited to MA including ≥10 studies. Available funnel plots are indicative of a publication bias for CAL and BOP scores and end-trial (Additional file 1: S11-20).

### Study outcomes

A table summarizing and presenting descriptive analysis of the statistical outcomes of the individual selected studies is provided in Additional file 1: S9. Additional file 1: S21 summarizes the outcome of the MA showing DiffM data between groups (SRP + amx + met versus SRP alone) at baseline and end of trial separately. Corresponding forrest plots are presented in Additional file 1: S22-37. The MA of the study outcomes of the treatment effect between groups, based on increments between baseline and end trial data are shown in Table 2. Corresponding forrest plots are presented in Additional file 1: S28-49. Underlying summaries and overviews of the selected studies with extracted outcome data of parameters of interest (PI,BOP,PD,CAL) are shown in Additional file 1: S56-59.

**Table 2:** Summary of the meta-analysis of the treatment effect between groups based on increments between baseline and end trial data (see Additional file 1 for further details)

Index # online supportive	ID# Selected studies	‘Random/Fixed’ effect model	Study duration	Difference in means between groups (in mm)	95 % confidence interval	*p*-value	Test for heterogeneity*	
Appendix	See Appendix S3					Test for overall effect	*p*-value	I^2^
Mean PD								
App. S38	IX, VIII, XIII	Fixed	Short term (2–3 months)	−0.49	(−0.6; −0.33)	<0.00001	0.31	14 %
	VI, XI, XVI, XVII		Medium term (6 months)	−0.41	(−0.57; −0.24)	<0.00001	0.49	0 %
	I, II, III		Long term (12 months)	−0.54	(−0.75; −0.34)	<0.00001	0.41	0 %
	All	Random	2–12 months	−0.47	(−0.58; −0.37)	<0.00001	0.57	0 %
PD > 4 mm								
App. S39	IV, V, X, XII, XIV	Random	3–24 months	−0.55	(−0.79; −0.30)	<0.0001	0.19	34 %
PD 4-6 mm								
App. S40	I, II, III, XVII, XIX	Random	6–12 months	−0.55	(−0.73; −0.37)	<0.00001	0.04	59 %
PD ≥ 6 mm								
App. S41	I, II, III, VI, VII, XII, XVII, XIX	Random	6–12 months	−0.86	(−1.07; −0.65)	<0.00001	0.51	0 %
Mean CAL								
App. S42	I, II, III, VI, VIII, IX, XI, XIII, XVI, XVII, XX	Random	3–24 months	+0.33	(0.23; 0.43)	<0.00001	0.83	0 %
CAL > 4 mm								
App. S43	IV, V, X, XII, XIV	Random	2–24 months	+0.35	(0.07; 0.63)	0.02	0.004	74 %
CAL 4-6 mm								
App. S44	I, II, III, XVII, XIX	Random	6–12 months	+0.42	(0.24; 0.61)	<0.00001	0.07	54 %
CAL ≥ 6 mm								
App. S45	I, II, III, VI, VII, XII, XVII, XVIII, XIX	Random	6–24 months	+0.75	(0.40; 1.09)	<0.0001	<0.0001	79 %

* = A chi-square test resulting in a *p* < 0.1 was considered an indication of significant statistical heterogeneity. As a rough guide for assessing the possible magnitude of inconsistency across studies, I2 statistic of 0–40 % was interpreted as not be important, and above 40 % moderate to considerable heterogeneity may be present

The overall analysis of the primary parameters of interest revealed that SRP + amx + met provided significantly better results regarding incremental differences in means (DiffM) of reduction in PD(DiffM:-0.47 mm, *p* < 0.00001) (Additional file 1: S38) and mean CAL gain (DiffM:+0.33 mm, *p* < 0.00001) (Additional file 1). The analysis for the secondary parameters showed that SRP + amx + met provided significantly better outcomes at end-trial regarding full mouth BOP (DiffM:-6.98 %, *p* = 0.0001) (Additional file 1: S47). With respect to the full mouth PI at end-trial there was no significant difference between SRP + amx + met compared to SRP alone(DiffM:-048, *p* = 0.68) (Additional file 1: S49).

Sub-analysis were performed for incremental changes in PD and CAL data based on initial probing depths at baseline. The analysis of change in PD at sites with baseline probing >4 mm showed significantly better effects for the SRP + amx + met group (DiffM:-0.55 mm, *p* = 0.0001) (Additional file 1: S39). Similarly sites with baseline PD 4–6 mm showed a significant difference between the SRP + amx + met group (DiffM:-0.55 mm, *p* < 0.00001) (Additional file 1: S40). Sites with baseline PD ≥6 mm also showed a significant difference between the SRP + amx + met and the SRP group (DiffM:-0.86, *p* < 0.00001) (Additional file 1: S41). Sub-analysis regarding the clinical attachment level (CAL) at sites with baseline PD >4 mm showed a significant incremental difference in favor of the SRP + amx + met group (DiffM:+0.35 mm, *p* = 0.02) (Additional file 1: S43). Similarly sites with baseline probing 4–6 mm (DiffM:+0.42 mm, *p* < 0.00001) (Additional file 1) and baseline pockets ≥6 mm (DiffM:+0.75 mm, *p* < 0.00001) (Additional file 1: S44) showed a significant gain in CAL. Sufficient data were available to perform a sub-analysis of PD in relation to study duration. Studies were sorted into short term (2–3 months), medium term (6 months) and long term (12 months). The SRP + amx + met group showed a significantly greater reduction as compared to SRP alone irrespective of the study duration (DiffM:-0.49 mm, −0.41and-0.54 respectively; test for subgroup differences *p* = 0.56) (Additional file 1: S38).

Sub-analysis was also performed based on the periodontal diagnosis as provided by the original papers. Table 3 shows the meta-analysis concerning the incremental differences between baseline and end-trial between groups. Additional file 1: S50-55 show that subgroups, divided into chronic, aggressive and unknown, follow a similar pattern of treatment effect. All in favour of the SRP + amx + met group.

**Table 3:** Summary of the meta-analysis of the treatment effect between groups based on increments between baseline and end trial data presented by subgroup analysis based on periodontal diagnosis (see Additional file 1 for further details)

Index # online supportive	ID# Selected studies	‘Random/Fixed’ effect model	Periodontal diagnosis	Difference in means between groups (in mm)	95 % confidence interval	*p*-value	Test for heterogeneity*	
Appendix	See Appendix S3					Test for overall effect	*p*-value	I^2^
Mean PD*								
App. S52	II,III,VI,VIII,XVI,XVII	Random	Aggressive Periodontitis	−0.48	(−0.33; −0.63)	<0.00001	0.32	14 %
	I,II,XIII		Chronic Periodontitis	−0.47	(−0.32; −0.63)	<0.00001	0.71	0 %
	NA		Unknown	NA	NA	NA	NA	NA
Mean CAL**								
App. S55	II,III,VI,VIII,XVI,XVII	Random	Aggressive Periodontitis	+0.39	(025; 0.53)	<0.00001	0.59	0 %
	I, IX. XIII		Chronic Periodontitis	+0.32	(0.16; 0.47)	<0.00001	0.64	0 %
	XX		Unknown	+0.00	(−0.35;0.35)	1.00	NA	NA

* = test for subgroup analysis *p* = 0.96

** = test for subgroup analysis *p* = 0.12

NA = not applicable

### Grading the body of evidence

Table 4 shows a summary of the various aspects that were used to rate the quality of the evidence and strength of the recommendations according to GRADE [28]. The data from the individual studies varied by parameter from rather consistent to inconsistent. The precision of the presented data was ‘precise’, the study outcomes were generalizable, and the magnitude of the effect was large in pockets initially ≥6 mm. All together the recommendation to prescribe a combination of amx + met concomitant to SRP was considered to be ‘strong’ for PD and ‘moderate’ for CAL based on the quality and body of evidence.

**Table 4:** Estimated evidence profile (GRADE, 2014) and appraisal of the strength of the recommendation

Determinants of the Quality		PPD mean	CAL mean
Study design		RCT, CCT	RCT, CCT
Risk of bias (methodological limitations)		Low to high	Low to high
Consistency		Rather consistent	Inconsistent
Directness		Generalizable	Generalizable
Precision		Precise	Precise
Reporting bias		Possible	Possible
Magnitude of the effect	Overall mean	Moderate	Moderate
	Pockets initially ≥ 6 mm	Large	Large
Strength of the recommendation based on the quality and body of evidence		Strong	Moderate

## Discussion

Antibiotics are effective means of treating bacterial infections and therefore constitute a reasonable consideration in the treatment of periodontal infections. A recent systematic review of concomitant administration systemic amoxicillin and metronidazole (amx + met) and SRP indicated the benefit of combination therapy. However, the review was limited by the absence of a comparison to SRP alone. Therefore this systematic review included studies with a direct comparison of SRP alone to SRP with adjunctive systemic amx + met. The aim of this SR was to evaluate in patients with periodontitis the available evidence concerning the effect of periodontal therapy including SRP + amx + met in comparison to SRP alone with respect to clinical parameters of periodontitis. Ultimately 20 clinical trials were selected (including a total number of 747 individual patients) from which data were obtained and used for the analysis. The key endpoint variable to evaluate the long-term efficacy of periodontal treatment preferably should be tooth survival. However due to the short study duration of the selected papers none have reported on this. Instead surrogate variables have been accepted as the main outcome measure, namely CAL and PD change [29].

The principle finding is that systemic amx + met therapy as adjunct to SRP significantly improved the clinical outcomes with respect to mean PD, CAL and BOP when compared to SRP alone. Superior clinical outcomes approximating a 1 mm difference for PD and CAL were observed especially in initially deep pockets (≥6 mm). This SR shows with respect to the primary outcomes of interest an improved reduction in overall mean PD of −0.47 mm (*p* < 0.00001) (Additional file 1: S38) and a mean additional gain in CAL of +0.33 mm (*p* < 0.00001) (Additional file 1: S42), both in favor of the SRP + amx + met. In those sites with a PD at baseline ≥6 mm the effect was even more pronounced with a difference in means between groups based on increments between baseline and end data for PD a DiffM of −0.86 (*p* < 0.00001) and for CAL a DiffM of +0.75 (*p* < 0.00001) (Additional file 1: S45). According to the parameters suggested by van Dyke [30] the results of these MA could be considered as clinically relevant. However it was not possible to investigate a generally accepted indicator for clinical relevance detection such as the percentage of sites that exhibit an improvement exceeding the threshold levels of 2 mm in PD or CAL [31].

The findings from this MA are more or less consistent with the results of previous SRs. The SR provided by Herrera et al. [2] showed a statistically significant additional effect of SRP + amx + met with regard to CAL change of 0.45 mm for sites with an initial PD >6 mm. The analysis of the treatment of aggressive periodontitis [32] resulted in a significant difference between groups in reduction in PD of −0.58 mm and gain in CAL of +0.42 mm in favor of the SRP + amx + met group. In a similar review evaluating the treatment effect in chronic periodontitis [33] a significant mean difference of +0.25 mm for the CAL gain and a −0.43 mm reduction PD in favor of the SRP + amx + met group was observed. Both reviews concluded that the findings appear to support the effectiveness of SRP + amx + met and that future studies are needed to confirm this results. Although the Sgolastra et al. reviews [32, 33] made a distinction between chronic and aggressive periodontitis a major concern in these reviews is the definition and classification of periodontitis. What signs and symptoms must be present in any specific individual to justify categorizing this specific individual as a ‘patient with periodontitis’ [34]? And when can periodontitis be specified as an aggressive or a chronic one. Following the classification of Van der Velden [35] one can distinguish between the different types of periodontitis based on patients’ age. According to this classification a criterion for post adolescent (aggressive) periodontitis is, when the age of the patient is between 21–35years. Periodontitis is classified as an adult (chronic), when the age is ≥36 years. Clearly from Additional file 1: S53 it can be seen that the inclusion in relation to age and diagnosis was stretched in the included papers. The distinction of the disease type in the studies included by Sgolastra et al. [32, 33] is not clear reflecting the change in the classification of periodontal diseases over time. Therefore it is debatable whether distinct differentiation between chronic and aggressive periodontitis truly reflects the patient populations of the included studies. Besides the two reviews also excluded studies for several reasons, e.g., lack of sample size calculation, randomization and allocation concealment methods, completeness of follow-up, presence of masking. Consequently, exclusion of potentially eligible studies that are performed with a proper methodology but poor reporting quality appears too strict. Some of the studies (Sgolastra et al. [32, 33] excluded) were identified and found suitable for inclusion in the present MA with the goal to be comprehensive for all available scientific evidence to support an evidence based treatment decision.

A previous review Zandbergen et al. [17] showed a potential effect for the antibiotic support when comparing the therapeutical effect data (baseline versus end-trial) to data as available from a SR from Van der Weijden&Timmerman [8] on SRP alone. The study outcomes were indicative of a clinical beneficial effect of SRP + amx + met suggesting that this combined therapy can enhance the effect of non-surgical periodontal therapy in healthy adults. The treatment effect as expressed as the full mouth weighted mean overall PD showed an improvement from baseline of 1.41 mm. The full-mouth weighted mean change for CAL showed a gain of 0.94 mm. However mean reduction in PD and mean gain in CAL may not be the best way to describe the present data. Shallow sites which are not expected to change as much as a result of the therapy [36] are likely to significantly dilute the changes observed at the deeper sites, which are the ones of therapeutic concern [37]. Therefore in addition a sub-analysis was performed on the change in PD and CAL based on a division in baseline PD. These data show that with respect to clinical outcome measures, treatment appeared to be strongly related to initial probing depth as was also observed by Van der Weijden&Timmerman [8].

As secondary outcomes PI and BOP were used (Additional file 1: S46-49). The analysis for the secondary parameters showed that SRP + amx + met provided significantly better effects regarding full mouth BOP (DiffM:-6.98 %, *p* = 0.0001) (Additional file 1: S47) in favor of the test group. There was no significant difference between SRP + amx + met and SRP alone with the respect to the full mouth PI (DiffM:-0.48, *p* = 0.68) (Additional file 1: S49). Reasonably this can be explained by the fact that most of the included studies started their therapy with an oral hygiene instruction (see Additional file 1: S7). Furthermore considering that the level of oral hygiene was comparative in both treatment groups, the additional mean reduction of PD and gain in CAL in favor of SRP + amx + met group gains in importance. The improved reduction in periodontal inflammation is also reflected by the secondary parameter BOP.

This SR focused on the additional benefit of SRP + amx + met compared to SRP alone. There is considerable evidence in support of SRP as an essential and effective component of therapy for the inflammatory periodontal diseases [36]. Periodontitis is a bacterial infection capable of enhancing the secondary host response and best described as an example of dysbiosis. A rationale for the use of adjunctive antimicrobial therapy is to help the human body to return to a state of symbiosis. Thereby antimicrobial therapy can have an additional effect at sites poorly influenced by mechanical therapy [4]. Nowadays it is known that antibiotics must always be used in conjunction with mechanical therapy to side step the protective effect of biofilm [12]. Attempts to eliminate subgingival bacteria without prior mechanical debridement to disrupt biofilm does not make sense [2]. However at which time during mechanical therapy the agent must be administered has not yet been completely defined [12]. In this context it is intriguing that elementary pharmacological studies on drug distribution demonstrated that inflammation, in general, can facilitate drug diffusion into various compartments of the body since perfusion and the permeability of capillaries are increased because of the hyperdynamic inflammatory state. In addition inflammatory hypoalbuminemia can decrease the degree of protein binding of antibiotics, which in turn results in increased concentration of the free drug [38–40]. This would suggest that administration of the antibiotics at the early stage of treatment will enhance the treatment effect as has also been shown by Griffiths et al. [41].

### Adverse events, bacterial resistance

However even though the treatment outcome with the amx + met is considerably enhanced, a precautionary restrictive attitude toward using antibiotics has been recommended [43]. Herrera et al. [3] stated that the risk of using antimicrobials should lead to a restriction in their use in periodontitis in certain patients and certain conditions, although a description of these is not provided. Conversely, adverse events (Additional file 1: S8), although not infrequent, were mild. Due to the risk for the development of adverse effects including gastrointestinal intolerance and hypersensitivity systemic antibiotics as an adjunct to periodontal therapy should be limited to patients with a high risk for disease progression [44].

In addition there is the general fear that the administration of a systemic antibiotic may lead to the emergence of “new” antibiotic resistant species. In the worst scenario these genes could encode information on resistance giving rise to a new bacterial population resistant to the agent in question. However there seems to be no major side effects associated with the intake of amx + met and indirect data suggest that increased proportions of antibiotic resistant species in the subgingival biofilm appear to occur largely as a result of selection of organisms that were naturally resistant to the antibiotic prior to antibiotic administration [45]. Proposed strategies to reduce the risk of bacterial antimicrobial resistance include prescribing two drugs with synergistic or complementary effect and administration of antibiotics at a high dose for a short period [46]. This strategy assumes that multiple species can be simultaneously eliminated or suppressed during periodontal therapy which leads to better stability of the microbiota and the host response and takes advantage of different specificities of the use of amx + met as a useful regimen with increase bactericidal and spectral efficacy when compared to mono therapy with each drug [47]. The true contribution to the resistance problem by the dentist treating a periodontal infection in a controlled situation following thorough mechanical debridement by administering two antibiotics with different antimicrobial action concomitantly is unknown and warrants future research. This contribution however, may be comparatively small in relation to the effect of the sometimes-indiscriminate consumption of antibiotics for other therapeutic and prophylactic reasons; dental and non-dental in nature [43]. Also under circumstances when a concomitant periodontal infection is not diagnosed by the physician, nor being treated before drug administration. The frequency and potential consequences of the unwanted systemic effects of antibiotics have to be balanced against the potential health consequences of not suppressing a periodontal infection quickly [43]. To balance to trade risks against benefits to the patient, benefits that could not be otherwise achieved or which would be achieved with much greater difficulty or risk by other means [45].

### Compliance

In addition subject compliance with unsupervised usage of the prescribed medication is critical [48]. Many factors have been related to a lack of adherence, misunderstanding of guidelines, gastrointestinal adverse events and/or duration of medication regimen [49]. The compliance of patients with the antibiotic intake has been scarcely reported in the selected studies (Additional file 1: S7). It is clear that non-compliance could undermine the true efficacy of the agent [2]. Reversely patients from countries with high prescription rates and low compliance exhibit more resistant bacteria than patients from countries with a low antibiotic consumption, a finding that has also been obtained for periodontal bacteria [50, 51]. The severest criticisms of the indiscriminate use of systemic antibiotics targets the side effects of the medications and particularly the development of bacterial resistance. The use of systemic antibiotics in a responsible manner, whenever their real efficacy for the treatment of a certain infection has been proved, is the best way of dealing with this [12]. The methods used to assess compliance such as patient self-report, interviews and counting tablets intake are not always objective and reliable. Especially self-reporting could therefore overestimate the results (32,33).

### Quality of studies and dosage

The included studies used different dosages and administration regimens in the SRP + amx + met group. A sub-analysis of the influence of dosage of AMX/MET on the clinical outcomes could not be performed due to the limited number of included studies for the different dosage groups. It is not possible to state whether such differences could have influenced the clinical outcomes. Additional file 1: S41 does show that the two studies with the largest treatment effect [34, 37, 52] are those with the higher dosage of AMX/MET. Because dosage is paramount in determining the microbiological and clinical outcomes of adjunctive systematic antimicrobial therapy, future studies are needed to assess the optimal dosage relative to the occurrence of adverse events and patient adherence to the treatment protocol [32, 33].

### Cost effectiveness

A cost/effectiveness analysis could not be performed because it was not reported by any of the included studies. Assessment of the cost/effectiveness ratio should include the risk of antimicrobial resistance, adverse events as well as the long-term prognosis. The costs-benefits ratio represents an important issue for clinicians and patients. The SRP + amx + met most probably will reduce the need for future nonsurgical treatment sessions. If however the downstream benefit is the elimination of the need for surgery, the interest of the patient and clinician are in conflict. Not doing surgery benefits the patient in terms of time, money and quality of life. Not doing surgery is a lost opportunity for income for the dentist. These competing value systems may have an impact on antibiotic use (or non-use).

### Limitations

This review has various limitations. Drug dosage, plaque control trial design, length of follow-up, disease severity and activity of the patient populations under investigation differ among studies and are important factors that should be taken in consideration [3]. Furthermore the heterogeneity regarding the antibiotics, daily dosage and length of drug regimens makes terminating conclusions about use in clinical practice difficult [40]. The possible impact of a publication bias on exaggerating the size of the test treatment effect should also be considered when interpreting the results. This systematic review narrowed down on a specific combination of two antibiotics and comprehensively evaluated the available evidence. In 3 recent systematic reviews [53–55] that evaluated systemic antibiotics in the treatment of periodontitis in a more broader sense come to the conclusion that out of all available antibiotics this combination is a most potent antibiotic combination and resulted in clinical improvements that were more pronounced. Limitations are further discussed in detail in Additional file 1: S60.

### Practical implication

Current periodontal therapy relies on primarily of meticulous mechanical supra- and subgingival debridement of tooth surfaces, which is ineffective in altering the oral microbiome. Conversely, met + amx is effective precisely because it alters the oral microbiome from dysbiosis to eubiosis. The use of systematic drugs could be therefore beneficial when used as adjuncts to conventional surgical and non-surgical therapy. The additional potential benefits could also contribute to improved systemic health. It may be emphasized that drugs, whether antimicrobials or host modulation agents, should not be used as a mono-therapy for the management of periodontal disease.

## Conclusions

The results of the meta-analysis performed in the present SR indicate that despite the caveats concerning the heterogeneity of experimental designs there is moderate to strong evidence that SRP + amx + met shows significantly superior clinical outcomes in terms of PD and CAL (especially in initially deep pockets; ≥6 mm) compared to SRP alone. Therefore it would seem correct to state that these agents are important allies in the treatment of periodontal infections. SRP + amx + met might therefore reduce the need for additional periodontal therapy which would assumedly be of a surgical nature in many cases. No major adverse events associated with the intake of amx + met were reported.

Some aspects of systemic antibiotics need future research. For instance, important issues are: what is the optimum dosage and duration to prescribe, which subjects benefit most from systematic antibiotics, when is the best time to start with the antibiotics during the debridement cycle and how long should we expect the administration to provide a clinical useful outcome. Besides, with the awareness of the side effects antibiotics must be prescribed in a responsible manner in order to avoid indiscriminate use which could lead to an increase in bacterial resistance.

## Additional file

Additional file 1:
**Online Supportive Appendices (additional file S61: [** 55 **–** 77 **]).** (DOC 1276 kb)

## Abbreviations

SRP: Scaling and root planing
amx: Amoxicillin
met: Metronidazole
PD: Pocket depth
CAL: Clinical attachment level
BOP: Bleeding on probing
PI: Plaque index
RCT: Randomized controlled trial
CCT: Controlled clinical trial
DZ: Dina Zandbergen; author of this paper
GAW: (Gode) Fridus August van der Weijden; author of this paper
SR: Systematic review
DES: Dagmar Else Slot; author of this paper
SD: Standard deviation
SE: Standard error
MA: Meta analysis
DiffM: Differences in means
