# Supplementary material for: The concomitant administration of systemic amoxicillin and metronidazole compared to scaling and root planing alone in treating periodontitis: =a systematic review=
Source: BMC Oral Health. 2016 Feb 29;16:27. doi: 10.1186/s12903-015-0123-6 (PMC4770674; doi:10.1186/s12903-015-0123-6)
Supplement: Additional file 1: — Online Supportive Appendices (additional file S61: [ 55 – 77 ]). (DOC 1276 kb) [file 12903_2015_123_MOESM1_ESM.doc]

**The Clinical Effect of Scaling and Root Planing and The**

**Concomitant Administration of Systemic Amoxicillin and Metronidazole Compared to Scaling and Rootplaning alone:**

**=A Systematic Review**=

**D. Zandbergen, D.E. Slot, R. Niederman, G.A. Van der Weijden**

**Online Supportive Appendices**

**Online Supporting information legends**

**Online Appendix S1** Search and Selection Results

**Online Appendix S2** Overview of the excluded studies

**Online Appendix S3** Study outline of selected papers

**Online Appendix S4** Characteristics of Study Design

**Online Appendix S5** Characteristics of the Participants

**Online Appendix S6** Smoking status

**Online Appendix S7** Characteristics of Interventions

**Online Appendix S8** Characteristics of Adverse Events

**Online Appendix S9** Descriptive Analysis

**Online Appendix S10** Quality assessment; methodological quality scores of the included studies

**Online Appendix S11** Funnel plot PD mean baseline scores; representing a symmetrical funnel suggesting absence of bias

**Online Appendix S12** Funnel plot PD mean end scores; representing a symmetrical inverted funnel, which is indicative of a data set in which publication bias is unlikely

**Online Appendix S13** Funnel plot CAL mean baseline scores; representing a symmetrical funnel suggesting absence of bias

**Online Appendix S14** Funnel plot CAL mean end scores; representing an a-symmetrical funnel indicating a potential risk of bias

**Online Appendix S15** Funnel plot differences mean PD scores. Sub-analysis of study duration, short-term (2-3 months), medium term (6 months), and long term (12 monhts); representing a symmetrical funnel suggesting absence of bias

**Online Appendix S16** Funnel plot differences mean CAL scores; representing a symmetrical funnel suggesting absence of bias

**Online Appendix S17** Funnel plot BOP baseline scores; representing a symmetrical funnel suggesting absence of bias

**Online Appendix S18** Funnel plot BOP end scores; representing an a-symmetrical funnel indicating a potential risk of bias

**Online Appendix S19** Funnel plot PI baseline scores; representing a symmetrical funnel suggesting absence of bias

**Online Appendix S20** Funnel plot PI end scores; representing an a-symmetrical funnel indicating a potential risk of bias

**Online Appendix S21** Meta-analysis of the baseline and end trial data

**Online Appendix S22** Forest plot of mean PD baseline scores; showing no significant difference between groups

**Online Appendix S23** Forest plot of mean PD scores at end of trial; showing a significant difference between groups following the intervention

**Online Appendix S24** Forest plot of PD baseline scores of sites with initial PD > 4 mm; showing no significant difference between groups

**Online Appendix S25** Forest plot of PD end scores of sites with initial PD > 4 mm; showing a significant difference between groups following the intervention

**Online Appendix S26** Forest plot of PD baseline scores of sites with initial PD 4-6 mm; showing a significant difference between groups

**Online Appendix S27** Forest plot of PD end scores of sites with initial PD 4-6 mm; showing a significant difference between groups following the intervention

**Online Appendix S28** Forest plot of PD baseline scores of sites with initial PD ≥ 6 mm; showing no significant difference between groups

**Online Appendix S29** Forest plot of PD end scores of sites with initial PD ≥ 6 mm; showing a significant difference between groups following the intervention

**Online Appendix S30** Forest plot of mean CAL at baseline; showing no significant difference between groups

**Online Appendix S31** Forest plot mean CAL scores at end trial; a significant difference between groups is observed where the loss of clinical attachment level is less in the experimental group

**Online Appendix S32** Forets plot of CAL baseline scores of sites with initial PD > 4 mm; showing no significant difference between groups

**Online Appendix S33** Forest plot of CAL end scores of sites with initial PD > 4 mm; a significant difference between groups is observed where the loss of clinical attachment level is less in the experimental group

**Online Appendix S34** Forest plot of CAL baseline scores of sites with initial PD 4-6 mm; showing no significant difference between groups

**Online Appendix S35** Forest plot of CAL end scores of sites with initial PD 4-6 mm; a significant difference between groups is observed where the loss of clinical attachment level is less in the experimental group

**Online Appendix S36** Forest plot of CAL baseline scores at sites with initial PD ≥ 6 mm; showing no significant difference between groups

**Online Appendix S37** Forest plot of CAL end scores of sites with initial PD ≥ 6 mm; a significant difference between groups is observed where the loss of clinical attachment level is less in the experimental group

**Online Appendix S38** Forest plot of the treatment effect (PD) between groups (random effects) based on increments between mean baseline and end data. Sub- analysis (fixed effects) of study duration, short term (2-3 months), medium term (6 months) and long term (12 months); showing all a significant difference between groups following the intervention

**Online Appendix S39** Forest plot of the treatment effect (PD) between groups based on increments between baseline and end data of sites with initial PD > 4 mm; showing a significant difference between groups following the intervention

**Online Appendix S40** Forest plot of the treatment effect (PD) between groups based on increments between baseline and end data of sites with intial PD of PD 4-6 mm; showing a significant difference between groups following the intervention

**Online Appendix S41** Forest plot of the treatment effect (PD) between groups based on increments between baseline and end data of sites with initial PD ≥ 6 mm; showing a significant difference between groups following the intervention

**Online Appendix S42** Forest plot of the treatment effect (CAL) between groups based on increments between mean baseline and end data; indicates that the gain in clinical attachment level is significant smaller in the control group than in the experimental group

**Online Appendix S43** Forest plot of the treatment effect (CAL) between groups based on increments between baseline and end data of sites with initial PD > 4 mm; indicates that the gain in clinical attachment level is significant smaller in the control group than in the experimental group

**Online Appendix S44** Forest plot of the treatment effect (CAL) between groups based on increments between baseline and end data of sites with initial PD 4-6 mm; indicates that the gain in clinical attachment level is significant smaller in the control group than in the experimental group

**Online Appendix S45** Forest plot of the treatment effect (CAL) between groups based on increments between baseline and end data of sites with initial PD ≥ 6 mm; indicates that the gain in clinical attachment level is significant smaller in the control group than in the experimental group

**Online Appendix S46** Forest plot BOP scores at baseline; showing no significant difference between groups

**Online Appendix S47** Forest plot BOP scores at end trial; showing a significant difference between groups following the intervention

**Online Appendix S48** Forest plot PI scores at baseline; showing no significant difference between groups

**Online Appendix S49** Forest plot PI scores at end trial; showing no significant difference between groups

**Online Appendix S50:** Forest plot PD scores at baseline in a subgroup analysis using the reported periodontal diagnosis as differentiation between groups

**Online Appendix S51:** Forest plot PD scores at end trial in a subgroup analysis using the reported periodontal diagnosis as differentiation between groups

**Online Appendix S52:** Forest plot PD scores, increment between baseline and end trial, in a subgroup analysis using the reported periodontal diagnosis as differentiation between groups

**Online Appendix S53:** Forest plot CAL scores at baseline in a subgroup analysis using the reported periodontal diagnosis as differentiation between groups

**Online Appendix S54:** Forest plot CAL scores at end trial in a subgroup analysis using the reported periodontal diagnosis as differentiation between groups

**Online Appendix S55:** Forest plot CAL scores, increment between baseline and end trial, in a subgroup analysis using the reported periodontal diagnosis as differentiation between groups

**Online Appendix S56** Summary and overview of selected studies. Outcome data extraction with respect to parameters of interest.

- Plaque scores

**Online Appendix S57** Summary and overview of selected studies. Outcome data extraction with respect to parameters of interest.

- Bleeding scores

**Online Appendix S58** Summary and overview of selected studies. Outcome data extraction with respect to parameters of interest.

- Mean probing pockets depth

**Online Appendix S59** Summary and overview of the selected studies. Outcome data extraction with respect to parameters of interest

- Mean clinical attachment level

**Online Appendix S60** Limitations of this systematic review

**Online Appendix S61** Additional references included in the Appendices and not provided in the main document

**Appendix S1: Search and Selection Results**

PubMed-MEDLINE

**260**

Cochrane-CENTRAL

**181**

EMBASE

**322**

**Identification**

Early view

**1**

**Screening**

Excluded by title and abstract

**509**

Unique title and abstract **573**

Selected papers for full-text reading

**64**

**Eligibility**

Excluded after full reading

**33**

Included from the reference list

**0**

Final Selection Papers

**31**

**Included**

Papers reporting on same study experiment

**2x1,3x1,4x1,2x1,**

**2x1,2x1,3x1**

Final Selection Studies

**20**

**Analysed**

**PI**

**9**


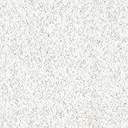


**BOP**

**14**


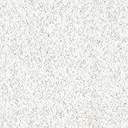


**mean CAL**

**10**


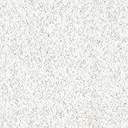


**mean PPD**

**11**


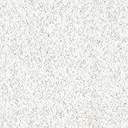


**Appendix S2: Overview of the excluded studies**

| **Author(s), (year)** | **Reason for rejection** |
| --- | --- |
| Ciancio (1993)  Bonito et al. (2004)  Baldan & Freeman (1991) | Narrative review |
| Herrera et al. (2002)  Herrera et al. (2008) | Systematic review |
| Rodrigues et al. (2012)  Valenza et al. (2009)  Johnson et al. (2008)  Guerrero et al. (2007)  Muller et al. (1998)  Winkel et al. (1998)  Winkelhoff van et al. (1992) | Cohort study |
| Akinincibay et al. (2008)  Machtei & Younis (2008)  Moreira & Feres-Filho (2007) | Lacking a control group |
| Kaner et al. (2007)  Beliveau et al. (2012) | Retrospective study  Retrospective study + SRP versus AB + participants too young |
| Dannewitz et al. (2007)  Buchmann et al. (2000)  Buchmann et al. (2002)  Tinoco et al. (1998) | Surgical periodontal therapy |
| López et al. (1998)  López et al. (2000)  López et al. (2006) | No SRP |
| Gaggl et al. (2006)  de Graaff (1989)  Kaner (2007)  Griffiths (2011) | Inappropriate data presentation |
| Pahkla et al. (2006) | SRP not in conjunction with systemic antibiotics |
| Rooney et al. (2002)  Serino et al. (2001)  Pavicic et al. (1994)  Winkelhoff van et al. (1989) | (Partly) Maintenance care patients |

**Appendix S3: Study outline of selected papers**

| **Study selection ID#**  **Author (year)**  **Title** | **Design & evaluation period** | **Diagnosis,**  **#subjects (end),**  **gender,**  **age** | **Regimen as adjunct to SRP** | **Original authors’ Conclusion** |
| --- | --- | --- | --- | --- |
| **I**  Soares et al. (2014)(28)  Feres et al. (2012)(29) | RCT  Parallel  Double-blind  12 months | Chronic  Periodontitis  79 (79)  ♀: 50 ◊  ♂: 29 ◊  Mean: 46.0 ◊  Range: NR | Amoxicillin 500 mg  3 x day  +  Metronidazole 400 mg  3 x day  14 days  Complement:  Rinsing with CHX or placebo | Treatment of generalized chronic periodontitis is significantly improved by the adjunctive use of amx+met. |
| **II**  Silva-Senem (2013)(30)  Heller et al. (2011)(31)  Varela et al. (2011)(32) | RCT  Parallel  Double-blind  12 months | Aggressive periodontitis  35 (35)  ♀: 22 ◊  ♂: 9 ◊  Mean: 32.6 ◊  Range: 18-39 | Amoxicillin 500 mg  3 x day  +  Metronidazole 250 mg 3 x day  10 days  Complement:  Pocket irrigation with CHX  Rinsing with CHX  Brushing dorsum tongue with CHX | amx+met brought additional clinical effects to the repeated mechanical and antiseptic treatment of generalised aggressive periodontitis (GAP), in the short-term (3 months), which have a tendency to fade away over time (6 months).  The enhanced anti-infective mechanical therapy is comparable with its combination with systemic amx+met for most clinical parameters and for maintaining low levels of periodontal pathogens for up to 1 year after treatment of GAP. |
| **III**  Lira et al. (2013)(33)  Mestnik et al. (2012)(34)  De Lima Oliveira et al.  (2012)(35)  Mestnik et al.(2010)(36) | RCT  Parallel  Double-blind  12 months | Aggressive periodontitis  30 (24)  Female: 14 ◊  Male: 10 ◊  Mean: 26.5 ◊  Range: NR | Amoxicillin 500 mg  3 x day  +  Metronidazole 400 mg  3 x day  14 days  Complement:  Rinsing with CHX | The non-surgical treatment of GAP is markedly improved by the adjunctive use of amx+met, up to 1 year post-treatment. |
| **IV**  Mombelli et al. (2013)(37) | RCT  Parallel  Double-blind  3 months | Moderate to advanced periodontitis  82 (82)  Female: 41  Male: 41  Mean: 48.3◊  Range: 25-70 | Amoxicillin 375 mg  3 x day  +  Metronidazole 500 mg  3 x day  7 days  Complement:  Pocket irrigation with CHX  Rinsing with CHX | In patients with moderate to advanced periodontitis systemic amx+met significantly enhanced the effects of full-mouth SRP, hereby reducing the need for further therapy, which according to current treatment concepts would be frequently surgical in nature. |

| **V**  Goodson et al. (2012)(38) | RCT  Parallel Multicenter  Single-blind  24 months | Diagnosis: NR  49 (49)  Female : NR  Male: NR  Mean: 47 ◊  Range: NR | Amoxicillin 500 mg  2 x day  +  Metronidazole 250 mg  3 x day  14 days  Complement:  Rinsing with CHX | A significant difference was established at 6 months and an improved CAL gain of 0.5 mm was maintained at 24 months. |
| --- | --- | --- | --- | --- |
| **VI**  Aimetti et al. (2012)(39) | RCT  Parallel  Double-blind  6 months | Aggressive periodontitis  39 (39)  ♀: 21 ◊  ♂: 18 ◊  Mean: 35.9 ◊  Range: NR | Amoxicillin 500 mg  3 x day  +  Metronidazole 500 mg  3 x day  7 days  Complement:  Pocket irrigation with CHX  Rinsing with CHX  Brushing dorsum tongue with CHX  Spray pharynx and tongue with CHX  Repeating subgingival application of CHX, 8 days after completion of the SRP | amx+met as an adjunct to one stage full-mouth disinfection therapy significant improved clinical outcomes in patients with GAP over a 6-month period. |
| **VII**  Casarin et al. (2012)(40) | RCT  Parallel  Double-blind  6 months | Aggressive periodontitis  25 (24)  Female: 16 ◊  Male: 8 ◊  Mean: 28.6 ◊  Range: NR | Amoxicillin 375 mg  3 x day  +  Metronidazole 250 mg  3 x day  7 days  Complement:  Sodium dipyrone only if needed as a painkiller | amx+met improves clinical results of full-mouth ultrasonic debridement in GAP patients. |
| **VIII**  Baltacioğlu et al. (2011)(41) | Pilot study  Parallel  Blinding ?  2 months | Aggressive periodontitis  26 (26)  ♀: 13  ♂: 13  Mean: 30.2 ◊  Range: 19-41 | Amoxicillin 250 mg  3 x day  +  Metronidazole 250 mg  3 x day  10 days | Treatment of GAP with full mouth root planning combined with systemic antibiotics provided significant clinical benefits that reduced the need for periodontal surgery. |
| **IX**  Silva et al. (2011)(42) | RCT  Parallel  Double-blind  3 months | Chronic  periodontitis  34 (34)  ♀: 19  ♂: 15  Mean: 47.2 ◊  Range: NR | Amoxicillin 500 mg  3 x day  +  Metronidazole 400 mg  3 x day  14 days | The adjunctive use of amx+met offers short-term clinical benefits in the treatment of non-smokers subjects with generalized chronic periodontitis. |
| **X**  Cionca et al. (2010) (43)  Cionca et al. (2009)(44) | RCT  Parallel  Double-blind  6 months | Moderate to advanced periodontitis  51 (47)  ♀: 30  ♂: 17 ◊  Mean: 50.5 ◊  Range: NR | Amoxicillin 375 mg  3 x day  +  Metronidazole 500 mg 3 x day  7 days  Complement:  Pocket irrigation with CHX  Rinsing with CHX | Systemic amx+met significantly improved the 6-month clinical outcomes of full-mouth non-surgical periodontal debridement, thus significantly reducing the need for additional therapy. |
| **XI**  Yek et al. (2010)(45) | RCT  Parallel  Single blind  6 months | Aggressive periodontitis  32 (28)  ♀: 19  ♂: 9  Mean: 31.0 ◊  Range: 15-45 | Amoxicillin 500 mg  3 x day  +  Metronidazole 500 mg 3 x day  7 days | Combined amx+met use as an adjunct to scaling and root planing leads to better clinical healing compared to mechanical treatment alone. |
| **XII**  Del Peloso Ribeiro et al. (2009)(46) | RCT  Parallel  Double-blind  6 months | Severe chronic periodontitis  28 (25)  ♀: 18 ◊  ♂: 7 ◊  Mean: 46.1 ◊  Range: 30-66 | Amoxicillin 375 mg  3 x day  +  Metronidazole 250 mg 3 x day  7 days | Both treatments resulted in significant clinical improvements; there was a slight, but significantly greater, improvement in bleeding on probing and a reduction in the percentage of sites with PD ≥ 5 mm exhibiting RAL gain ≥ 2 mm in the test group. |
| **XIII**  Matarazzo et al.  (2008)(47) | RCT  Parallel  Double-blind  3 months | Chronic periodontitis  30 (29)  ♀: 16  ♂: 13  Mean: 41.6 ◊  Range: NR | Amoxicillin 500 mg  3 x day  +  Metronidazole 500 mg  3 x day  14 days | Significant advantages are observed when systematic antibiotics are combined with SRP in the treatment of smokers with chronic periodontitis. The greatest benefits in clinical parameters are achieved with the use of SRP+amx+met. |

| **XIV**  Moeintaghavi et al. (2007)(47) | RCT  Parallel  Double blind  2 months | Moderate to severe chronic periodontitis  NR (50)  ♀: 29  ♂: 21  Mean: 34.4 ◊  Range: 17-51 | Amoxicillin 500 mg  3 x day  +  Metronidazole 250 mg  3 x day  7 days  Complement:  Vitamin B preparations | The significant differences are in line with other studies and support the considerable adjunctive benefits of the combination of amx+met in the treatment of chronic periodontitis. |
| --- | --- | --- | --- | --- |
| **XV**  Giannopoulou et al. (2006)(8)  Mombelli et al. (2005)(13) | RCT  Parallel  (partial mouth)  Double blind  12 months | Chronic periodontitis  16 (14)  ♀: 6  ♂: 10  Mean: NR  Range:25-65 | Amoxicillin 375 mg  3 x day  +  Metronidazole 250 mg  3 x day  7 days  Complement:  Pocket irrigation with a saline solution | Improved healing of the soft tissues has been noted clinically in non-surgically treated sites in subjects treated with antibiotics. |
| **XVI**  Xajigeorgiou et al. (2006)(49) | RCT  Parallel  Single blind  6 months | Aggressive periodontitis  23 (21)  ♀: 10  ♂: 11  Mean: 37.9 ◊  Range: 22-49 | Amoxicillin 500 mg  3 x day  +  Metronidazole 500 mg  3 x day  7 days | Adjunctive amx+met is effective in deep pockets of GAP patients. |
| **XVII**  Guerrero et al. (2005)(50)  Guerrero et al. (2014)(61) | RCT  Parallel  Double-blind  6 months | Aggressive periodontitis  41 (41)  ♀: 28  ♂: 13 ◊  Mean: 31.5 ◊  Range: NR | Amoxicillin 500 mg  3 x day  +  Metronidazole 500 mg  3 x day  7 days  Complement:  Rinsing with CHX | A 7-day adjunctive course of systemic amx+met significantly improved the short- term clinical outcomes of full-mouth non-surgical periodontal debridement in subjects with GAP. |
| **XVIII**  Ehmke et al. (2003)(52)  Flemmig et al. (1998)(53)  Ehmke et al. (2005)(11) | RCT  Parallel  Blinding?  24 months | Moderate to severe chronic periodontitis  NR (35)  ♀: 19  ♂: 16 ◊  Mean: 51.0 ◊  Range: NR | Amoxicillin 375 mg  3 x day  +  Metronidazole 250 mg  3 x day  8 days  Complement:  Rinsing with CHX | Over the 24-month period, a single course of the administered adjunctive antimicrobial therapy led to a relative risk reduction of 62% for attachment loss at deep sites. |
| **XIX**  Winkel et al. (2001) (54) | RCT  Parallel  Double blind  6 months | Severe periodontitis  54 (49)  ♀: 28  ♂: 21  Mean: 42  Range: 28-63 | Amoxicillin 375 mg  3 x day  +  Metronidazole 250 mg  3 x day  7 days | Systemic usage of amx+met, when used in conjunction with initial periodontal treatment in adult periodontitis patients, achieves significantly better clinical results than initial periodontal treatment alone. |
| **XX**  Berglundh et al. (1998) (55) | RCT  Parallel  (partial mouth)  Blinding?  12 months | Advanced periodontal disease  Test:  16 (NR)  ♀: NR  ♂: NR  Mean:NR  Range: NR | Amoxicillin 375 mg  2 x day  +  Metronidazole 250 mg  3 x day  14 days | The combined mechanical and systemic antibiotic therapy was more effective than mechanical therapy alone in terms of improvement of clinical features of periodontal disease. |

GAP = Generalized Aggressive Periodontitis

MET = Metronidazole

AMX = Amoxicillin

PD = Pocketdepth

CAL = Clinical Attachment Level

RAL = Relative Attachment Level

SRP = Scaling and Root Planing

CHX = Chlorhexidine

NR = Not Reported

**Appendix S4: Characteristics of Study Design**

Of the selected studies, all were randomized controlled clinical trials. Except for one study, which was a pilot study (VIII). 16 studies measured full-mouth scores for the primary and secondary outcomes (I, II, III, V, VI, VIII, IX, X, XI, XIII, XIV, XVI, XVII, XVIII, XIX, XX). 14 of these studies scored at 6 sites per tooth (I, II, III, IV, VI, VIII, IX, X, XI, XIII, XVI, XVII, XVIII XIX). One study excluded teeth with pulpal disease or furcation lesions (VII) and one other study excluded malpositioned teeth (XIV). Four studies described that only indicator teeth or test sites were measured (IV, VII, XII, XV). The description of the indicator teeth or test sites varied between the studies. Two studies (XII, XX) mentioned a stent was used to define probing attachment loss (PAL) according to a standard protocol, for details see Westfelt et al. (1996). Some studies mentioned there was only one examiner for the measurements (II, III, IV, VII, XI, XII, XIII, XIV, XV, XVI, XVII, XVIII, XIX). From seven studies it was unknown whether the clinicians were calibrated (IV, VIII, X, XI, XV, XIX, XX). 13 studies mentioned calibration, (I, II, III, IV, V, VI, VII, XII, XIII, XIV, XVI, XVII, XVIII).

**Reference:**

Westfelt, E., Rylander, H., Blohmé, G., Jonasson, P. & Lindhe, J. (1996) The effect of periodontal therapy in diabetics. Results after 5 years. *Journal of Clinical Periodontology* **23**, 92-100.

**Appendix S5: Characteristics of the Participants**

Ten studies evaluated patients with chronic (adult) periodontitis (I, IV, IX, X, XII, XIII, XIV, XV, XVIII, XX) and nine studies included patients with aggressive periodontitis (II, III, VI, VII, VIII, XI, XVI, XVII, XIX). One study did not describe the diagnosis (V) Some studies specifically selected patients with subgingival infections exhibiting periodontopathogens such as *Aggregatibacter actinomycetemcomitans* (XVIII) or *Porghyromonas gingivalis* (XV). In the study of Mombelli et al. (2013) only patients were selected for the control group if they harboured *Aggregatibacter actinomycetemcomitans*. All studies enrolled patients untreated for periodontal disease or no definitive therapy within the previous 6 months. From one study (VIII) it was not reported if the participants were in good general health whereas all participants of the other selected studies were described to be in good general health.

**Reference:**

Mombelli, A., Cionca, N., Almaghlouth, A., Décaillet, F., Courvoisier, D. S. & Giannopoulou, C. (2013) Are there specific benefits of amoxicillin plus metronidazole in *Aggregatibacter actinomycetemcomitans*-associated periodontitis? Double-masked, randomized clinical trial of efficacy and safety. *Journal of Periodontology* **84**, 715-724. [selection ID: **IV]**

**Appendix S6: Smoking status**

Some studies included smokers. Some of these did not describe any details about the number of participants or the number of cigarettes they smoked (IV, X, XV, XVI, XVII). Other studies included smokers when the participants smoked at least 10 cigarettes a day for the last 5 years (XIII), when the participants were current smokers or quit smoking within the last year (XIX) or when they smoked less than 10 cigarettes a day (XI). The remaining studies provided details concerning the number and/or percentage of smokers among the participants, which varied between studies from 6% to 40% (II, V, VIII, XVII). From most of the studies it was indistinguishable if there was a difference in clinical data between the smokers and non-smokers, although one study did mention there was no difference in clinical data between the smokers and non-smokers (XVIII). One study mentioned that smoking reduced CAL gain and PPD reduction (V). Study (XVII) concluded that in a non-smoker, deep pockets reduced by an average of 0.9 mm more than in a smoker, regardless of the treatment group. One other study described that the reduction in PPD and gain of CAL was significantly less in the placebo/smokers subgroup compared to the test/smokers subgroup (XIX).

**Appendix S7: Characteristics of Interventions**

Most of the studies started their therapy with an oral hygiene instruction. The greater part of the studies did not provided details on the type of instruction given (I, II, III, IV, VII, VIII, IX, X, XI, XIII, XVI, XVII, XVIII, XIX, XX). Different studies instructed approximal cleaning (V, XIV), some specified this in dental floss and interdental brushes (VI, XII). One study instructed their participants to additionally brush the dorsum of the tongue (XI). One study instructed their participants the electric toothbrush and the use of triclosan containing toothpaste (V). Eleven studies performed a session of supragingival debridement ahead of the SRP to facilitate the dental hygiene (III, IV, VI, VII, VIII, IX, X, XII, XIII, XIX, XX). In most of the selected studies standard full-mouth periodontal therapy was provided consisting of SRP. One study only treated pockets ≥4 mm, (XV). One study (X) treated at least half of the diseased teeth and one other study treated only the periodontally diseased teeth (IV). Some studies complemented SRP with an irrigation of the pockets or advised the participants to rinse with a CHX solution, during or following the sessions of SRP (details are described in Appendix S3).

Most of the studies did not point out who had been responsible for the instrumentation. Ten studies mentioned that the treatment was completed by periodontists (I, II, III, IV, VI, X, XI XII, XVI, XVII). One study allowed senior dental students to perform the instrumentation (XVIII). Some studies described that the quality of SRP performed, was controlled by a supervisor like a periodontal resident (I, XVIII), a study coordinator (IX) or a senior investigator (II). The number of sessions planned for SRP varied among the studies from 1 session to 6 sessions. The combination of ultrasonic and manual instruments dominated the type of instruments used for SRP among the studies (II, IV, VI, X, XIV, XV, XVII). From (V, VIII, XI, XIII, XIX, XX) it was unknown what kind of instruments they used.

Regarding the combination of antimicrobial therapy administered adjunctive to the SRP, there were variations between the studies in both dosage and duration (for details see table appendix S3). The dosage for amoxicillin as well as for metronidazole varied from 250 mg to 500 mg among the studies. Most studies prescribed to take the antibiotics three times daily. The duration of medication varied among the studies from seven (IV, VI, VII, X, XI, XII, XIV, XV, XVI, XVII, XIX), eight days (XVIII), 10 days (II, VIII), to 14 days (I, III, V, IX, XIII, XX). The antimicrobial therapy was started in most of the studies immediately after the last session of SRP (IV, V, VI, VII, VIII, XII, XIV, XV, XVII, XVIII). Some studies started after the first session of SRP (I, II, III V, XI, XIII, XIX), one started during the first 2 weeks of active therapy (XX) and two studies started 6 weeks after the last session of SRP (XVI, XIX). Most studies did not evaluate the compliance of the medication intake. Six studies mentioned a compliance rate of 100% with a medication intake of 2 or 3 times a day (II, III, V, VI, XVI, XIII). One study mentioned a compliance rate of 76.92% (XII).

**Appendix S8: Characteristics of Adverse Events**

Two studies mentioned there were no serious adverse events (V, VIII). In five studies it was unknown if there were any adverse effects related to the systemic use of antimicrobial therapy (IX, X, XV, XVII, XX). Nausea, diarrhea, vomiting and gastrointestinal discomfort were the most common adverse events mentioned (I, II, III, IV, VI, VII, IX, XII, XIII, XIV, XVI, XIX). One study mentioned nausea after the use of alcohol (XIX). Oral manifestations described were a metallic taste (I, II, IX), taste alterations (II), ulcerations (II, VII), mouth burning, staining of the tongue and tooth (II). Other side effects described were fever (VII), dizziness (II), headache, irritability (I, IX) and a rash on the neck of the face (IX, XIX). Worth reporting is that participants in the placebo groups also complained about side effects such as nausea, diarrhea (II) vomiting (III), dizziness, oral ulcerations (II), and weakness (IX).

**Appendix S9: Descriptive Analysis**

| **Selected Study ID#** | **Intervention** | **PI** | **BOP** | **PD**  **mean** | **PD**  **> 4mm** | **PD**  **4-6 mm** | **PD**  **≥ 6mm** | **CAL**  **mean** | **CAL**  **> 4mm** | **CAL**  **4-6 mm** | **CAL**  **≥ 6mm** |
| --- | --- | --- | --- | --- | --- | --- | --- | --- | --- | --- | --- |
| **VIII** | **AMX 250 mg**  **MET 250 mg** | ○ | ○ | **+** | **□** | **□** | **□** | **+** | **□** | **□** | **□** |
| **VII** | **AMX 375 mg**  **MET 250 mg** | ○ | ○ | **□** | **□** | **□** | ○ | **□** | **□** | **□** | ○ |
| **XII** |  | ○ | **+** | **□** | **+** | **□** | ? | **□** | **+** | **□** | ? |
| **XV** |  | ○ | ? | **□** | **+** | **□** | **□** | **□** | **+** | **□** | **□** |
| **XVIII** |  | **□** | ? | **□** | **□** | ○ | **□** | **□** | **□** | **□** | **+** |
| **XIX** |  | ○ | **+** | **+** | **□** | **+** | **+** | **+** | **□** | ○ | **+** |
| **XX** |  | ○ | ? | ? | **□** | **□** | **□** | ? | **□** | **□** | **□** |
| **IV** | **AMX 375 mg**  **MET 500 mg** | ○ | ○ | **□** | **+** | **□** | **□** | **□** | **□** | **□** | **□** |
| **X** |  | ○ | ○ | **□** | + | **□** | **□** | **□** | ○ | **□** | **□** |
| **II** | **AMX 500 mg**  **MET 250 mg** | ○ | ○ | ○ | **□** | ○ | ○ | ○ | **□** | ○ | ○ |
| **V** |  | ? | ? | **□** | **+** | **□** | **□** | **□** | **+** | **□** | **□** |
| **XIV** |  | **+** | **+** | **□** | **+** | **□** | **□** | **□** | **+** | **□** | **□** |
| **I** | **AMX 500 mg**  **MET 400 mg** | ○ | ○ | **+** | **□** | **+** | **+** | **+** | **□** | **+** | **+** |
| **III** |  | ○ | ○ | **+** | **□** | **+** | **+** | ○ | **□** | **+** | **+** |
| **IX** |  | ○ | ○ | **+** | **□** | **□** | **□** | **+** | **□** | **□** | **□** |
| **VI** | **AMX 500 mg**  **MET 500 mg** | ○ | **+** | **+** | **□** | **□** | **+** | **+** | **□** | **□** | **+** |
| **XI** |  | ○ | **□** | ○ | **□** | **□** | **□** | ○ | **□** | **□** | **□** |
| **XIII** |  | ? | **+** | **+** | **□** | **□** | **□** | **+** | **□** | **□** | **□** |
| **XVI** |  | **□** | ○ | ○ | **□** | **□** | **□** | ○ | **□** | **□** | **□** |
| **XVII** |  | ○ | **+** | **+** | **□** | **+** | **+** | ○ | **□** | **+** | **+** |

**□ =** no data

○ = no significant difference

+ = significant difference in favor of test group

- = significant difference in favor of control group

**Appendix S10: Quality assessment; methodological quality scores of the included studies**

| **Validity** | Selected Study ID# | I | II | III | IV | V | VI | VII | VIII | IX | X | XI | XII | XIII | XIV | XV | XVI | XVII | XVIII | XIX | XX |
| --- | --- | --- | --- | --- | --- | --- | --- | --- | --- | --- | --- | --- | --- | --- | --- | --- | --- | --- | --- | --- | --- |
|  | Quality criteria |  |  |  |  |  |  |  |  |  |  |  |  |  |  |  |  |  |  |  |  |
| **External** | Representative population Group | + | + | + | + | + | + | + | + | + | + | + | + | + | + | + | + | + | + | + | + |
|  | Eligibility Criteria defined ♦ | + | + | + | + | + | + | + | + | + | + | + | + | + | + | + | + | + | + | + | + |
| **Internal** | Random  Allocation ♦ | + | + | + | + | + | + | + | ? | + | + | + | + | + | + | + | + | + | + | + | + |
|  | Allocation concealment | + | + | - | - | - | ? | + | - | + | ? | ? | + | + | ? | - | ? | + | - | - | - |
|  | Blinded to the patient ♦ | + | + | + | + | - | + | + | ? | + | + | - | + | + | + | + | - | + | ? | + | ? |
|  | Blinded to the examiner ♦ | + | + | + | + | + | + | + | ? | + | + | + | + | + | + | + | + | + | ? | + | ? |
|  | Blinding during statistical analysis | + | - | - | - | - | - | - | - | - | - | - | - | - | - | - | - | - | - | - | - |
|  | Balanced experimental groups | + | + | + | + | + | + | + | + | + | + | + | + | + | + | + | + | + | + | + | + |
|  | Reported loss to follow up ♦ | + | + | + | + | + | + | + | + | + | + | + | + | + | - | + | + | + | - | + | - |
|  | # (%) of drop-outs | 0  (0%)  ◊ | 0  (0%)  ◊ | 6 (20%)  ◊ | 0 (0%)  ◊ | 0 (0%)  ◊ | 0 (0%)  ◊ | 1 (8%)  ◊ | 0  (0%)  ◊ | 0  (0%)  ◊ | 4  (8%)  ◊ | 4  (12%)◊ | 3  (11%) ◊ | 1 (3%)  ◊ | ? | 2  (12%)  ◊ | 2  (9%)  ◊ | 0 (0%)  ◊ | ? | 5 (9%)  ◊ | ? |

|  | Treatment identical except for intervention | + | + | + | + | + | + | + | + | + | + | + | + | + | ? | + | + | + | + | + | + |
| --- | --- | --- | --- | --- | --- | --- | --- | --- | --- | --- | --- | --- | --- | --- | --- | --- | --- | --- | --- | --- | --- |
| **Statistical** | Sample size calculation and power | + | + | + | - | + | + | + | - | + | - | + | + | + | + | - | + | + | - | - | - |
|  | Point estimates Presented for primary  outcome ♦ | + | + | + | + | + | + | + | + | + | + | + | + | + | + | + | + | + | + | + | + |
|  | Measures of variability for the primary outcome | + | + | + | + | + | + | + | + | + | + | + | + | + | + | + | + | + | + | + | + |
|  | Unit of analysis | FM | FM | FM | Site | Site | FM | Site | FM | FM | FM | FM | FM | FM | FM | Site | FM | FM | FM | FM | FM |
|  | Reproducibility data shown/described | + | + | + | - | + | + | + | - | + | - | - | + | + | + | - | + | + | + | - | - |
|  | Intention to treat analysis | + | + | - | - | + | - | - | - | - | - | - | - | - | - | - | - | - | - | - | - |
|  | Per protocol analysis | - | - | + | + | - | + | + | + | + | + | + | + | + | - | - | + | + | + | + | ? |
|  |  |  |  |  |  |  |  |  |  |  |  |  |  |  |  |  |  |  |  |  |  |
|  | Estimated potential risk of bias | L | L | L | L | M | L | L | H | L | L | M | L | L | H | M | M | L | H | L | H |
|  | Oxford grades for level of evidence | 1b | 1b | 1b | 1b | 1b | 1b | 1b | 2b | 1b | 1b | 2b | 1b | 1b | 2b | 1b | 1b | 1b | 2b | 1b | 2b |

? = Not specified/unclear

+ = Yes

- = No

◊ = Calculated by the authors

FM = Full mouth

Site = Only test sites analyzed

± = Calibration mentioned, no data shown

♦ = Criteria for Quality Assessment

Each aspect of the score list was given a ‘+’ for an informative description of the item at issue for a study design meeting the quality standard, a ‘-‘ for an informative description, but a study design not meeting the quality standard and a ‘?’ for lacking or insufficient information. When random allocation, defined eligibility criteria, blinding of examiners, balanced experimental groups, identical treatment between groups (except for intervention) and report of follow up were present, the study was classified as having a low (L) risk of bias. When one of these six criteria was missing, the study was considered to have a moderate (M) potential risk of bias. When two or more of these criteria were missing, the study was considered to have a high (H) potential risk of bias, as proposed by Van der Weijden et al. ^xx^

**Appendix S11: Funnel plot PD mean baseline scores; representing a symmetrical funnel suggesting absence of bias**


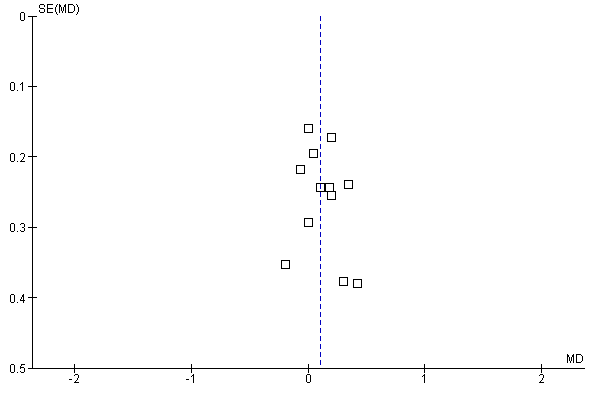


**Appendix S12: Funnel plot PD mean end scores; representing a symmetrical inverted funnel, which is indicative of a data set in which publication bias is unlikely**


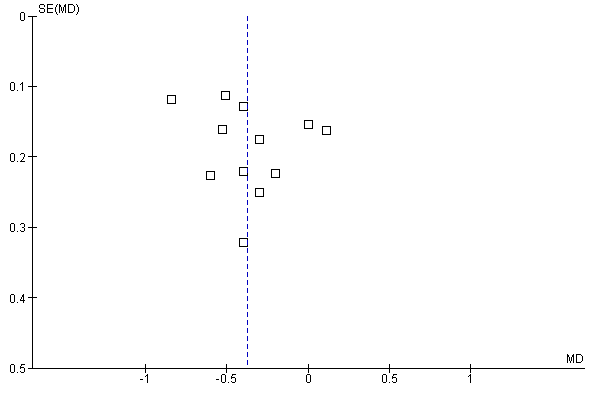


**Appendix S13: Funnel plot CAL mean baseline scores; representing a symmetrical funnel suggesting absence of bias**

**
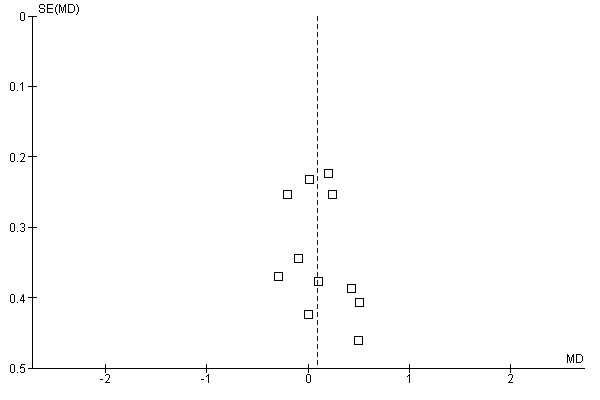
**

**Appendix S14: Funnel plot CAL mean end scores; representing an a-symmetrical funnel indicating a potential risk of bias**


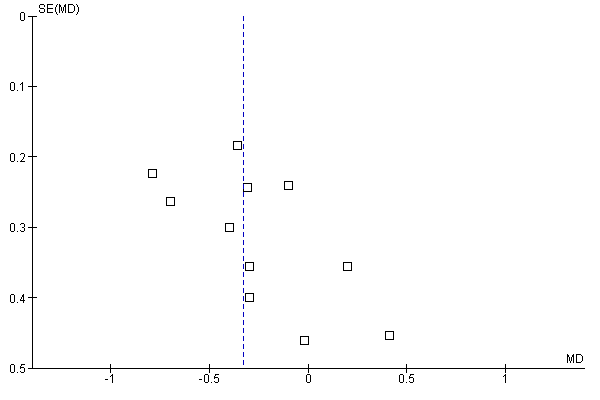


**Appendix S15: Funnel plot differences mean PD scores. Sub-analysis of study duration, short-term (2-3 months), medium term (6 months), and long term (12 monhts); representing a symmetrical funnel suggesting absence of bias**


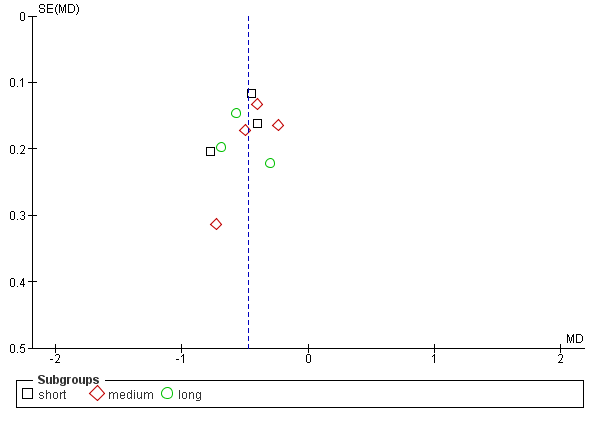


**Appendix S16: Funnel plot differences mean CAL scores; representing a symmetrical funnel suggesting absence of bias**


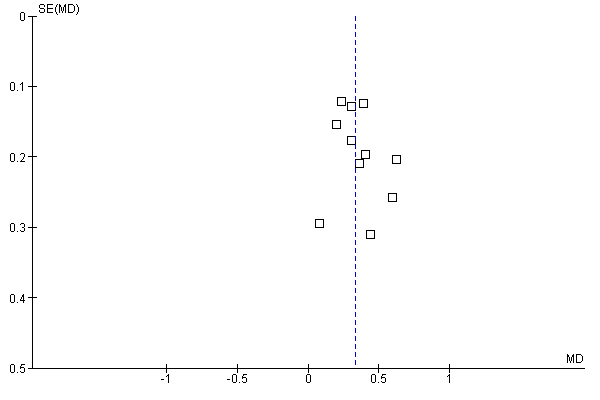


**Appendix S17: Funnel plot BOP baseline scores; representing a symmetrical funnel suggesting absence of bias**


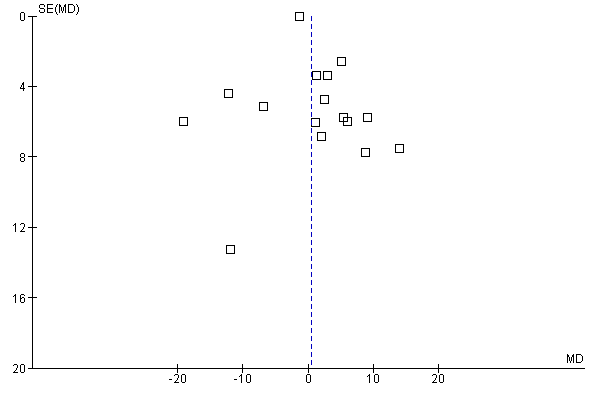


**Appendix S18: Funnel plot BOP end scores; representing an a-symmetrical funnel indicating a potential risk of bias**

**
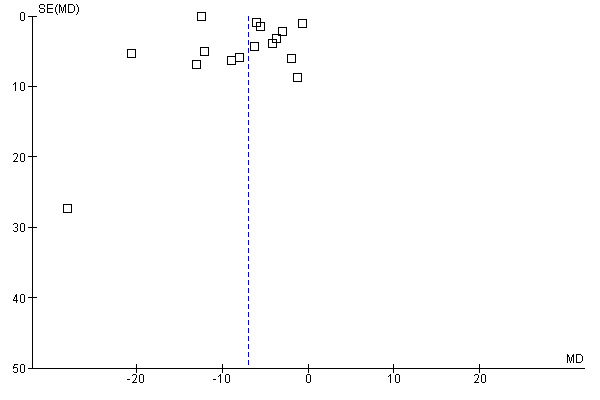
**

**Appendix S19: Funnel plot PI baseline scores; representing a symmetrical funnel suggesting absence of bias**


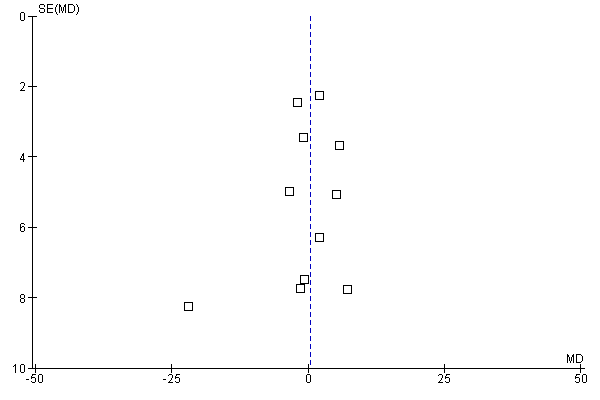


**Appendix S20: Funnel plot PI end scores; representing an a-symmetrical funnel indicating a potential risk of bias**


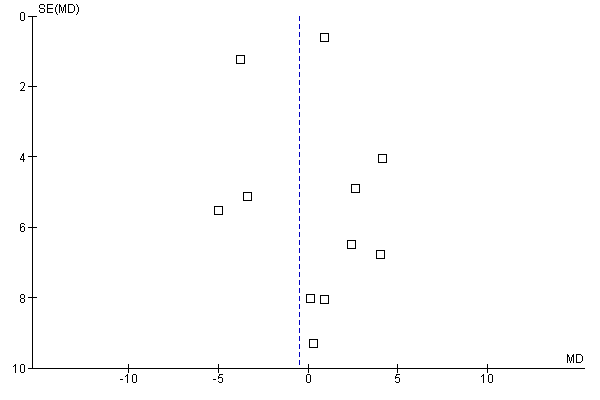


**Appendix S21: Meta-analysis of the baseline and end trial data**

| **Index**  **# online Supportive**  **Appendix** | **ID**  **Selected Studies**  **See Appendix S3** | **‘Random/**  **Fixed’ effect model** | **Study duration** | **Difference**  **in means**  **between**  **groups**  **(in mm)** | **95% confidence interval** | **p-value**  **Test for overall effect** | **Test for heterogeneity^*^** | |
| --- | --- | --- | --- | --- | --- | --- | --- | --- |
|  |  |  |  |  |  |  | **P-value** | **I^2^** |
| Plaque  App. S48,S49 | I, II, III, V,VI, VII, IX, XII, XIII, XVII, XX | Random | Base | **0.29** | (-2.69; 3.27) | 0.85 | 0.21 | 25% |
|  |  |  | End | **-0.48** | (-2.77; 1.80) | 0.68 | 0.15 | 32% |
| BOP  App. S46,S47 | I, II, III, V, VI, VII, VIII, IX, X, XII, XIV, XV, XVII, XVIII, XX | Random | Base | **0.43** | (-2.73; 3.59) | 0.79 | 0.0008 | 61% |
|  |  |  | End | **-6.98** | (-10.58; -3.38) | 0.0001 | <0.00001 | 94% |
| PD mean  App. S22,S23 | I, II, III, VI, VIII, IX, XI, XIII, XVI, XVII, XIX, XX | Random | Base | **0.11** | (-0.03; 0.24) | 0.11 | 0.95 | 0 % |
|  |  |  | End | **-0.37** | (-0.54; -0.20) | <0.00001 | 0.0003 | 68% |
| PD > 4mm  App. S24,S25 | IV, V, X, XII, XIV, XV | Random | Base | **0.04** | (-0.11; 0.19) | 0.61 | 0.65 | 0% |
|  |  |  | End | **-0.39** | (-0.59; -0.19) | 0.0001 | 0.09 | 48% |
| PD 4-6mm  App. S26,S27 | I, II, III, XVII | Fixed | Base | **0.07** | (0.02; 0.13) | 0.01 | 0.26 | 25% |
|  |  |  | End | **-0.56** | (-0.69; -0.42) | <0.00001 | 0.07 | 58% |
| PD ≥ 6mm  App. S28,S29 | I, II, III, VI, VII, XII, XVII | Random | Base | **-0.08** | (-0.22; 0.06) | 0.25 | 0.97 | 0% |
|  |  |  | End | **-0.91** | (-1.25; -0.57) | <0.00001 | 0.01 | 62% |
| CAL mean  App. S30,S31 | I, III, VI, VIII, XIII, XVII | Random | Base | **0.09** | (-0.09; 0.27) | 0.33 | 0.81 | 0% |
|  |  |  | End | **-0.33** | (-0.52; -0.14) | 0.0007 | 0.25 | 21% |
| CAL > 4mm  App. S32,S33 | II, IX, XI, XVI, XIX | Random | Base | **0.24** | (-0.01; 0.50) | 0.06 | 0.70 | 0% |
|  |  |  | End | **-0.10** | (-0.35; 0.16) | 0.47 | 0.55 | 0% |
| CAL 4-6mm  App. S34,S35 | I, II, III, XVII | Fixed | Base | **0.15** | (-0.04; 0.34) | 0.13 | 0.33 | 13% |
|  |  |  | End | **-0.41** | (-0.64; -0.18) | 0.0005 | 0.80 | 0% |
| CAL ≥ 6mm  App. S36,S37 | I, II, III, VI, VII, XII, XVII | Random | Base | **-0.12** | (-0.36; 0.13) | 0.35 | 1.00 | 0% |
|  |  |  | End | **-0.66** | (-1.12; -0.20) | 0.005 | 0.01 | 63% |

*=A chi-square test resulting in a p < 0.1 was considered an indication of significant statistical heterogeneity. As a rough guide for assessing the possible magnitude of inconsistency across studies, I2 statistic of 0–40% was interpreted as not be important, and above 40% moderate to considerable heterogeneity may be present.

**Appendix S22: Forest plot of mean PD baseline scores; showing no significant difference between groups**


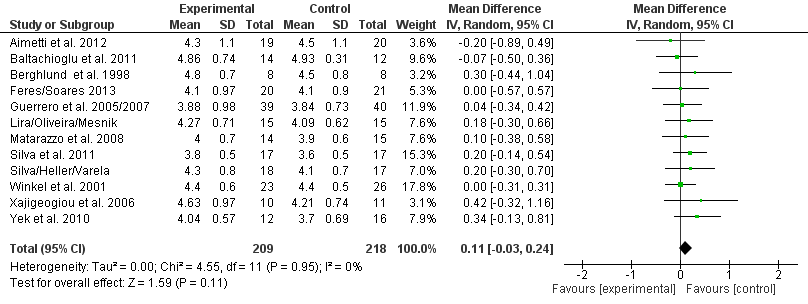


**Appendix S23: Forest plot of mean PD scores at end of trial; showing a significant difference between groups following the intervention**


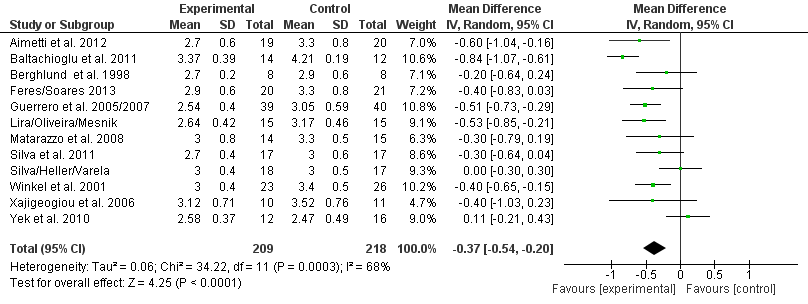


**Appendix S24: Forest plot of PD baseline scores of sites with initial PD > 4 mm; showing no significant difference between groups**


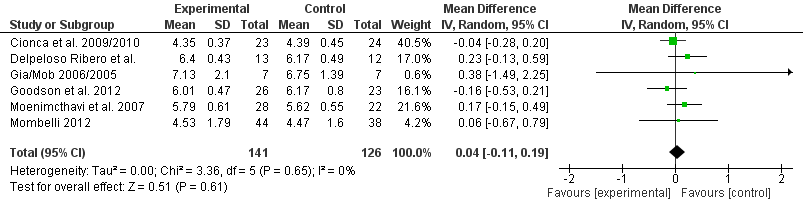


**Appendix S25: Forest plot of PD end scores of sites with initial PD > 4 mm; showing a significant difference between groups following the intervention**


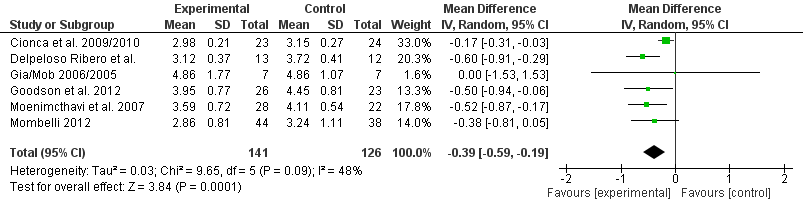


**Appendix S26: Forest plot of PD baseline scores of sites with initial PD 4-6 mm; showing a significant difference between groups**


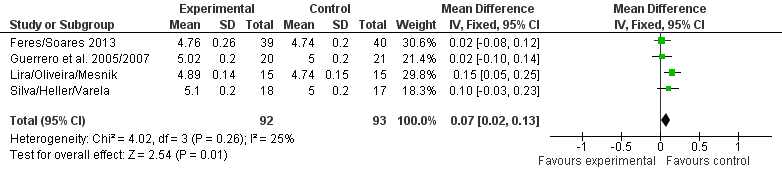


**Appendix S27: Forest plot of PD end scores of sites with initial PD 4-6 mm; showing a significant difference between groups following the intervention**


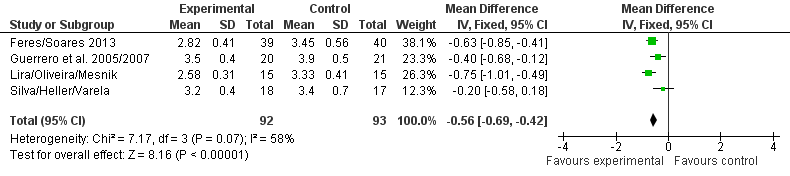


**Appendix S28: Forest plot of PD baseline scores of sites with initial PD ≥ 6 mm; showing no significant difference between groups**


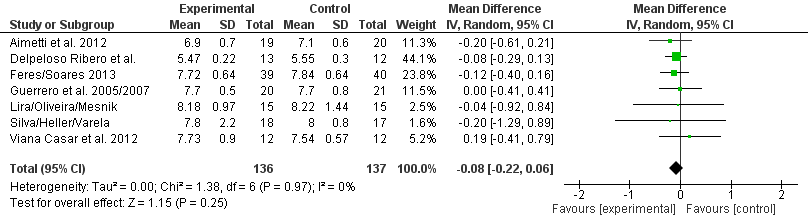


**Appendix S29: Forest plot of PD end scores of sites with initial PD ≥ 6 mm; showing a significant difference between groups following the intervention**


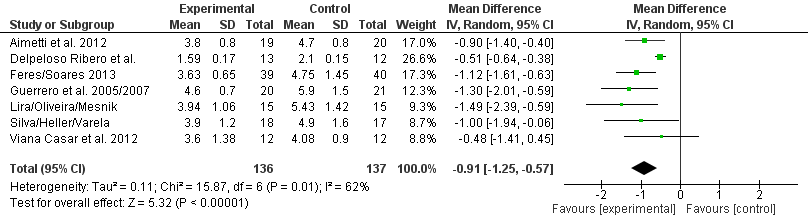


**Appendix S30: Forest plot of mean CAL at baseline; showing no significant difference between groups**


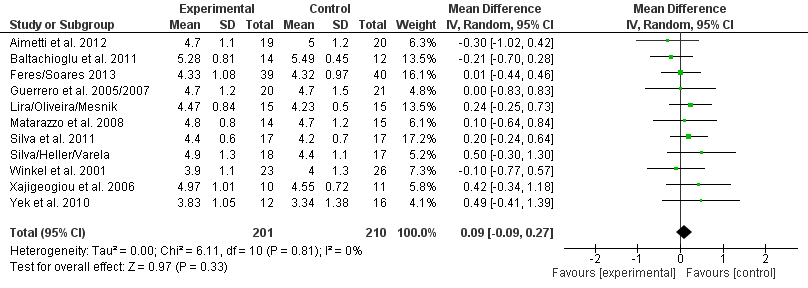


**Appendix S31: Forest plot mean CAL scores at end trial; a significant difference between groups is observed where the loss of clinical attachment level is less in the experimental group**


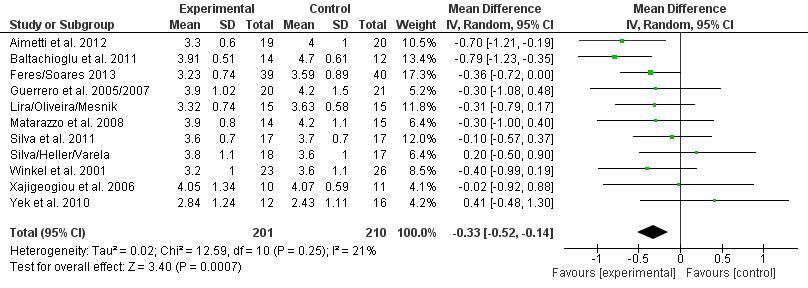


**Appendix S32: Forets plot of CAL baseline scores of sites with initial PD > 4 mm; showing no significant difference between groups**


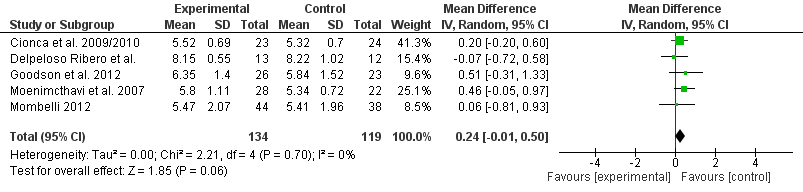


**Appendix S33: Forest plot of CAL end scores of sites with initial PD > 4 mm; a significant difference between groups is observed where the loss of clinical attachment level is less in the experimental group**


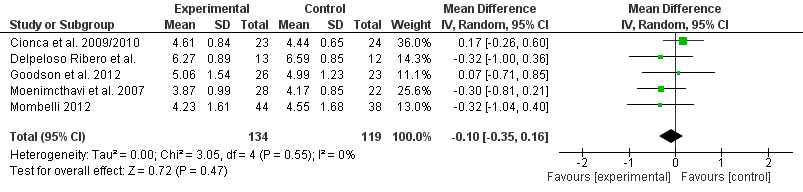


**Appendix S34: Forest plot of CAL baseline scores of sites with initial PD 4-6 mm; showing no significant difference between groups**


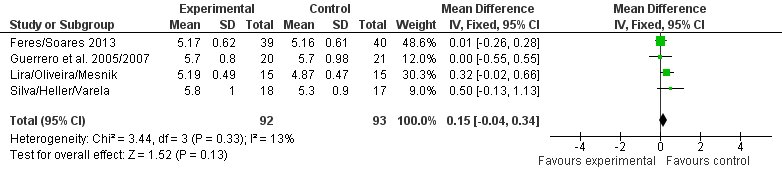


**Appendix S35: Forest plot of CAL end scores of sites with initial PD 4-6 mm; a significant difference between groups is observed where the loss of clinical attachment level is less in the experimental group**


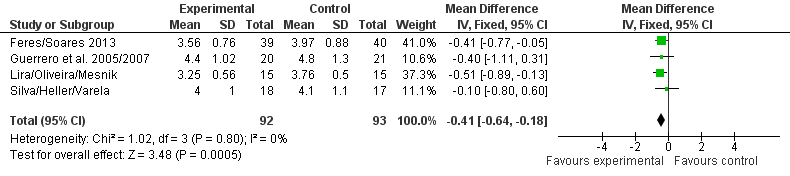


**Appendix S36: Forest plot of CAL baseline scores at sites with initial PD ≥ 6 mm; showing no significant difference between groups**


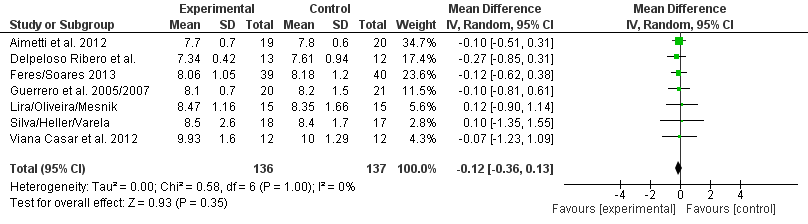


**Appendix S37: Forest plot of CAL end scores of sites with initial PD ≥ 6 mm; a significant difference between groups is observed where the loss of clinical attachment level is less in the experimental group**


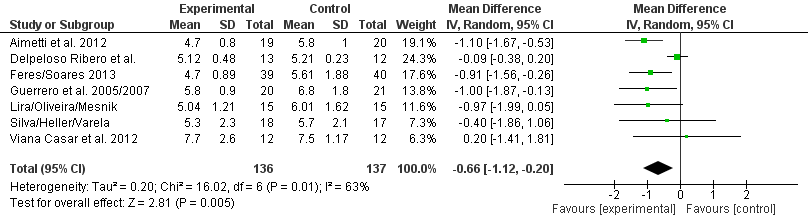


**Appendix S38: Forest plot of the treatment effect (PD) between groups (random effects) based on increments between mean baseline and end data. Sub- analysis (fixed effects) of study duration, short term (2-3 months), medium term (6 months) and long term (12 months); showing all a significant difference between groups following the intervention**


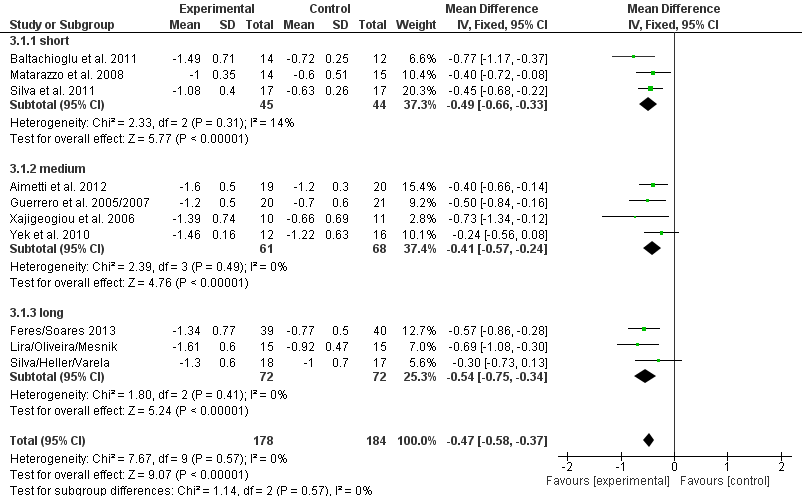


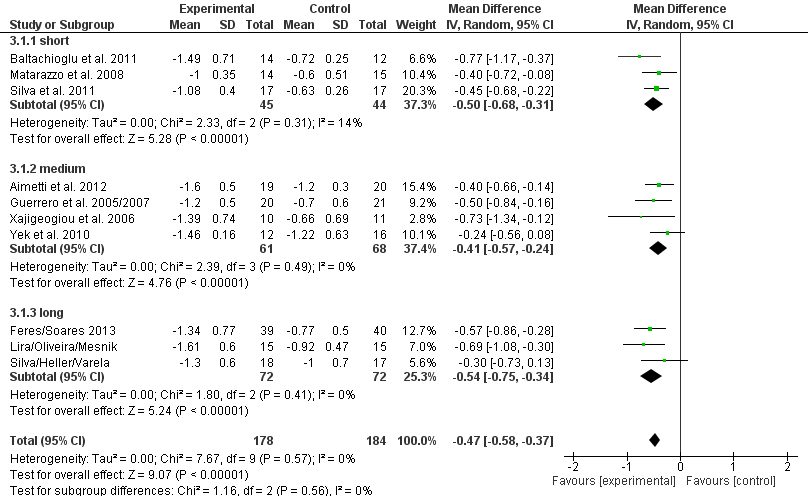


**Appendix S39: Forest plot of the treatment effect (PD) between groups based on increments between baseline and end data of sites with initial PD > 4 mm; showing a significant difference between groups following the intervention**


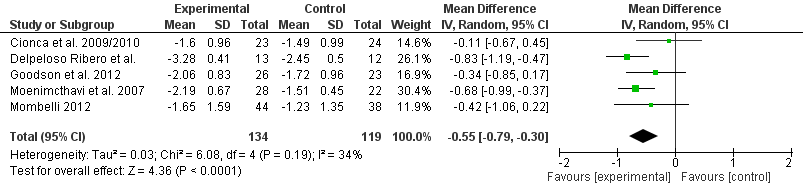


**Appendix S40: Forest plot of the treatment effect (PD) between groups based on increments between baseline and end data of sites with intial PD of PD 4-6 mm; showing a significant difference between groups following the intervention**


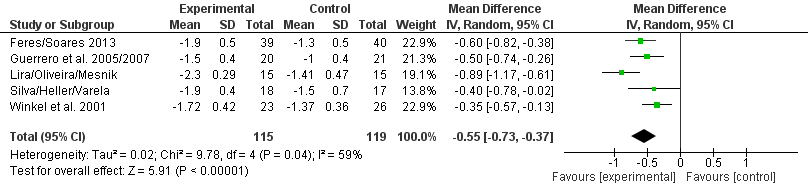


**Appendix S41: Forest plot of the treatment effect (PD) between groups based on increments between baseline and end data of sites with initial PD ≥ 6 mm; showing a significant difference between groups following the intervention**


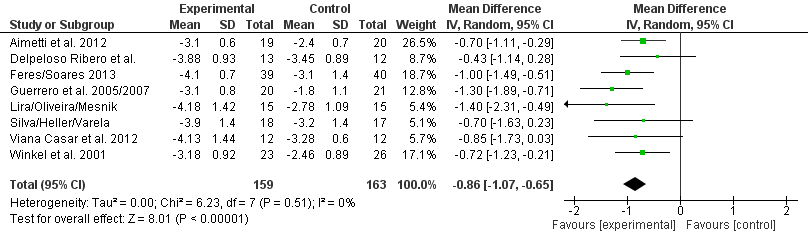


**Appendix S42: Forest plot of the treatment effect (CAL) between groups based on increments between mean baseline and end data; indicates that the gain in clinical attachment level is significant smaller in the control group than in the experimental group**


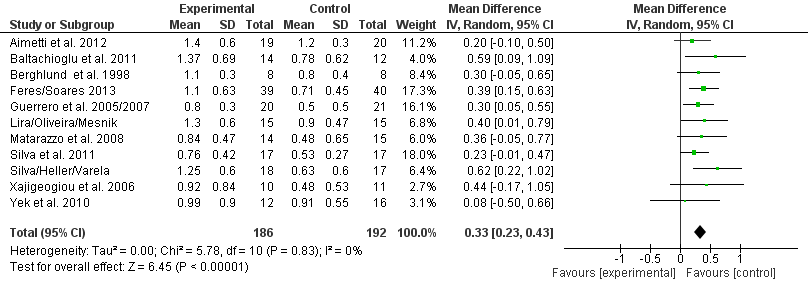


**Appendix S43: Forest plot of the treatment effect (CAL) between groups based on increments between baseline and end data of sites with initial PD > 4 mm; indicates that the gain in clinical attachment level is significant smaller in the control group than in the experimental group**


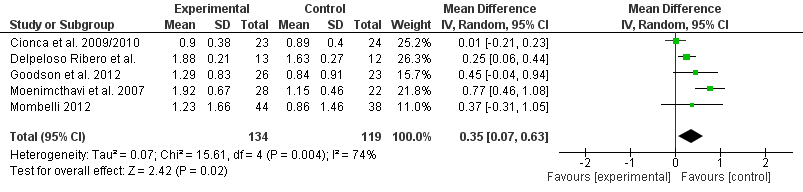


**Appendix S44: Forest plot of the treatment effect (CAL) between groups based on increments between baseline and end data of sites with initial PD 4-6 mm; indicates that the gain in clinical attachment level is significant smaller in the control group than in the experimental group**


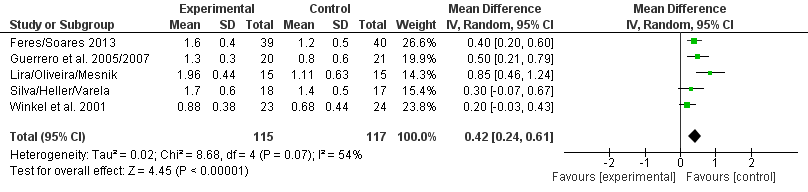


**Appendix S45: Forest plot of the treatment effect (CAL) between groups based on increments between baseline and end data of sites with initial PD ≥ 6 mm; indicates that the gain in clinical attachment level is significant smaller in the control group than in the experimental group**


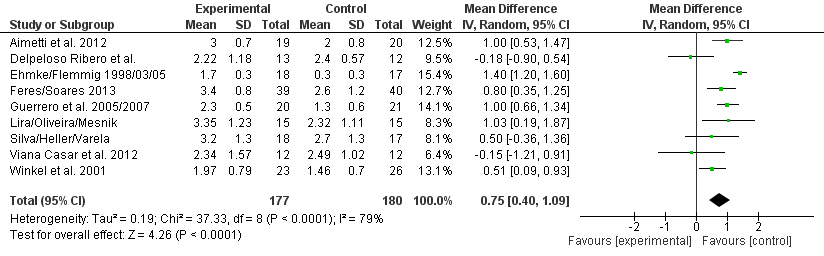


**Appendix S46: Forest plot BOP scores at baseline; showing no significant difference between groups**


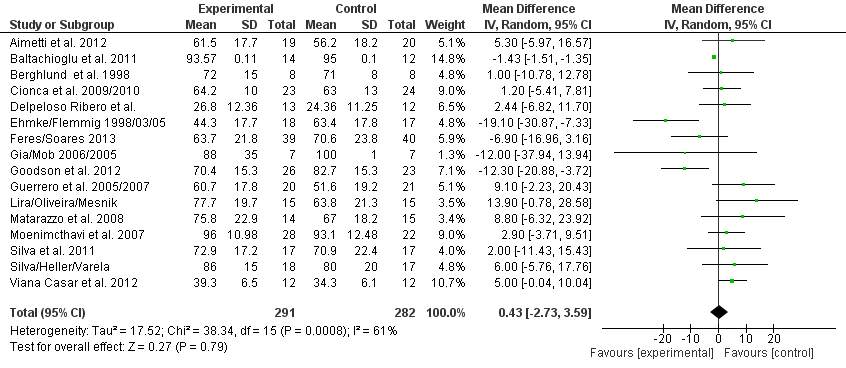


**Appendix S47: Forest plot BOP scores at end trial; showing a significant difference between groups following the intervention**


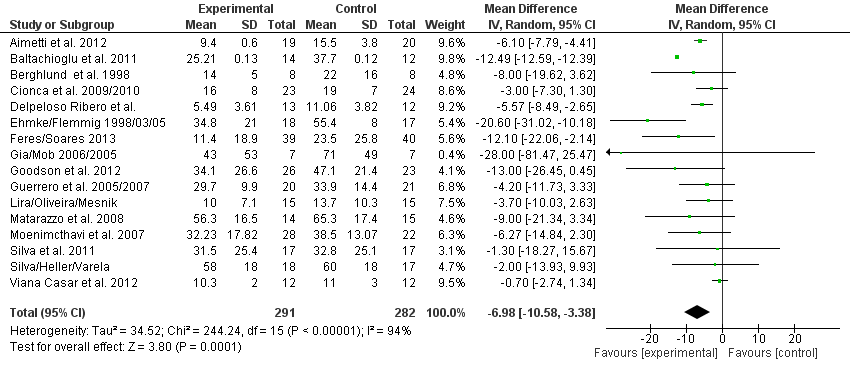


**Appendix S48: Forest plot PI scores at baseline; showing no significant difference between groups**


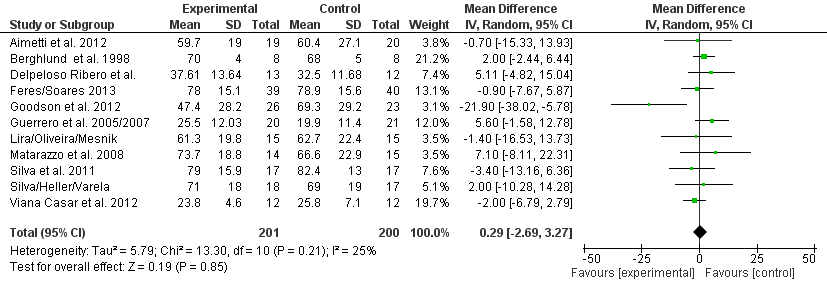


**Appendix S49: Forest plot PI scores at end trial; showing no significant difference between groups**


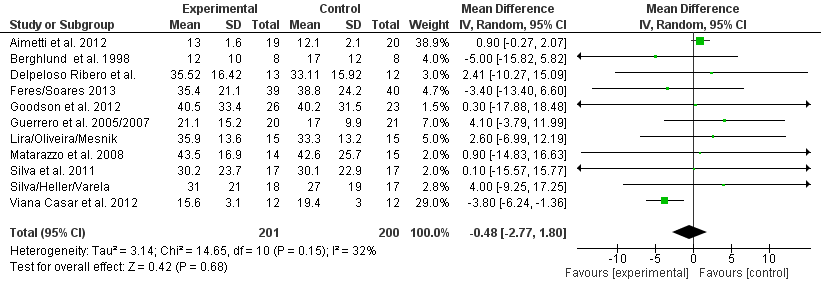


**Appendix S50: Forest plot PD scores at baseline in a subgroup analysis using the reported periodontal diagnosis as differentiation between groups**


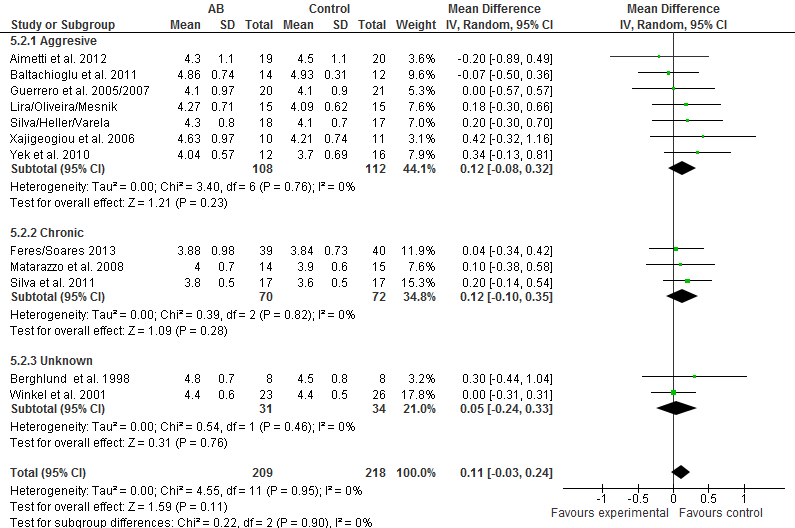


**Appendix S51: Forest plot PD scores at end trial in a subgroup analysis using the reported periodontal diagnosis as differentiation between groups**


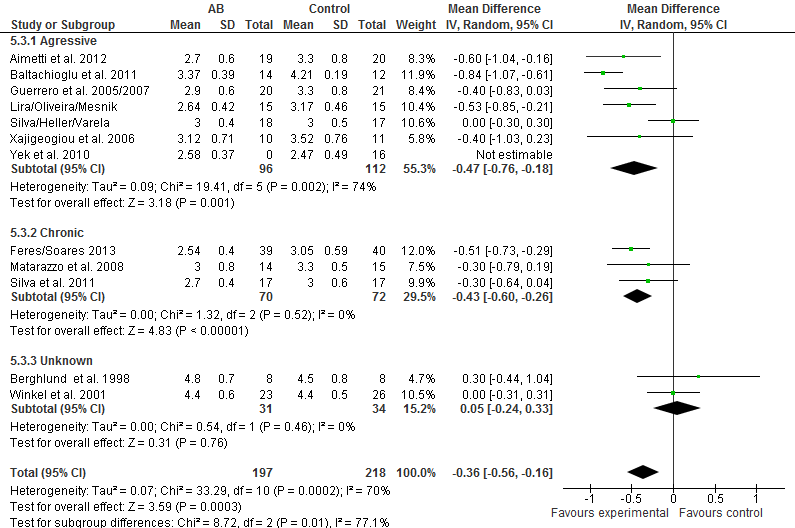


**Appendix S52: Forest plot PD scores, increment between baseline and end trial, in a subgroup analysis using the reported periodontal diagnosis as differentiation between groups**


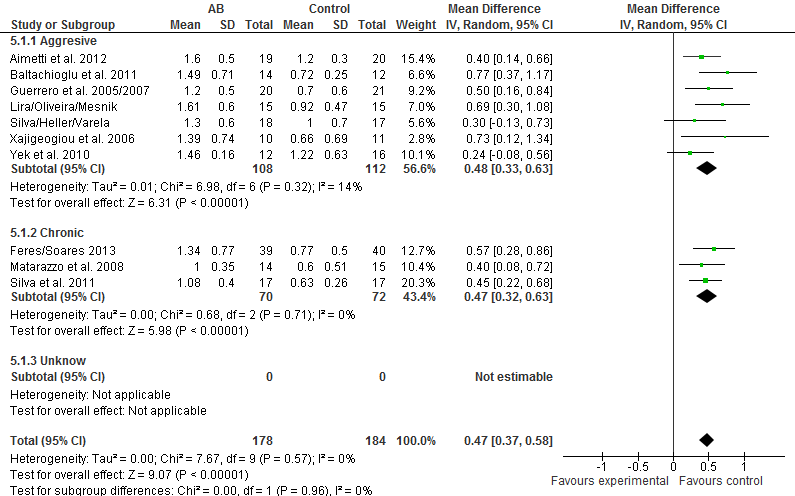


**Appendix S53: Forest plot CAL scores at baseline in a subgroup analysis using the reported periodontal diagnosis as differentiation between groups**


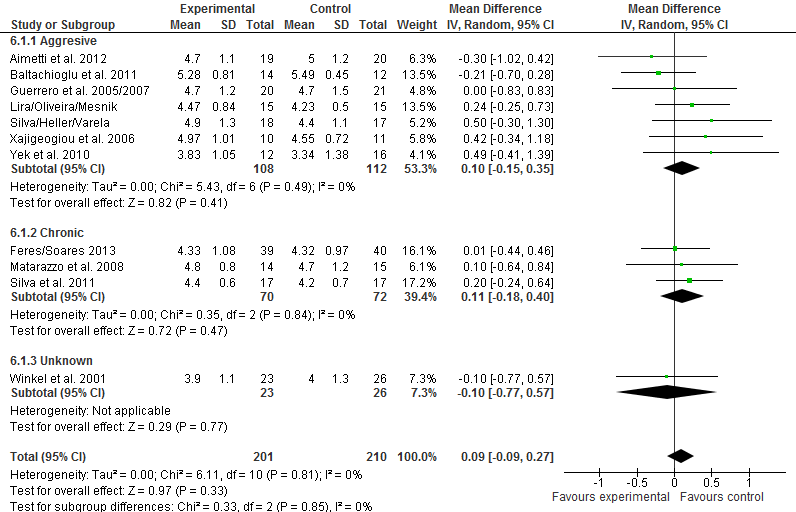


**Appendix S54: Forest plot CAL scores at end trial in a subgroup analysis using the reported periodontal diagnosis as differentiation between groups**


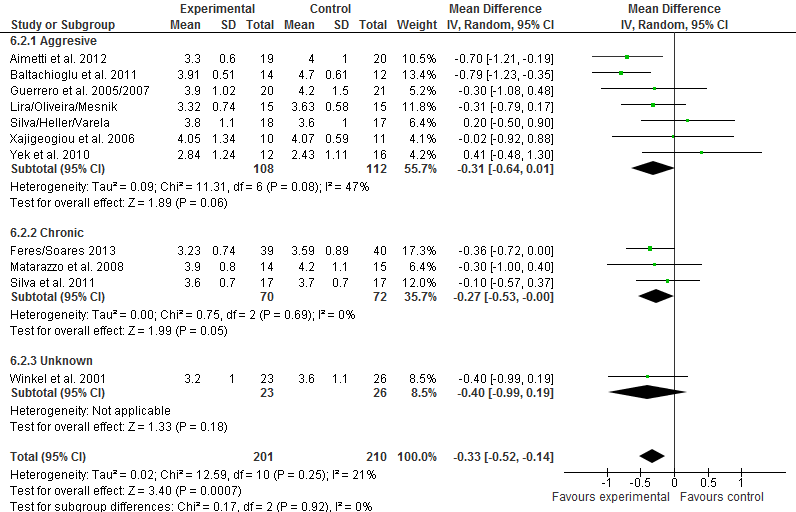


**Appendix S55: Forest plot CAL scores, increment between baseline and end trial, in a subgroup analysis using the reported periodontal diagnosis as differentiation between groups**


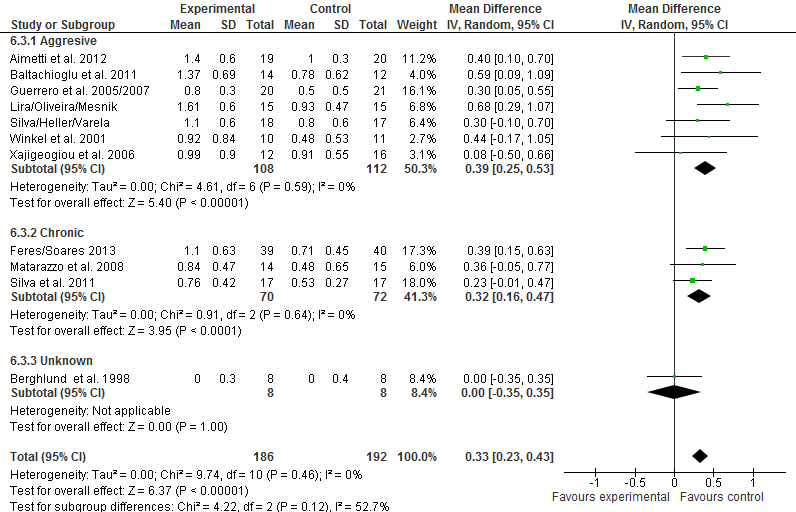


**Appendix S56: Summary and overview of selected studies. Outcome data extraction with respect to parameters of interest.**

**Plaque scores**

| **Value** | **ID# of selected studies** | | | **Index** | **Test/**  **Control**  **group** | **Mean**  **Baseline**  **scores** | **Mean**  **End**  **scores** | **Difference**  **(absolute)** | **Significant**  **(within group)** | **Difference**  **(relative %)** | **N** | **Evaluation**  **Period** |
| --- | --- | --- | --- | --- | --- | --- | --- | --- | --- | --- | --- | --- |
| **Mean** | IV | | | Plaque score * | Test | 0.54 (0.5) ♦ | 0.35 (0.49) ♦ | -0.19 (0.59) ♦ | ? | -35 ◊ | 44 | 3 months |
|  |  |  |  |  | Control | 0.42 (0.49) ♦ | 0.30 (0.46) ♦ | -0.12 (0.55) ♦ | ? | -29 ◊ | 38 | 3 months |
|  | VIII | | | PI  Silness & Loë 1964 | Test | 1.81 (0.75) | 0.20 (0.29) | -1.61 (0.88) ♦ | Yes | -89 ◊ | 14 | 2 months |
|  |  |  |  |  | Control | 1.85 (0.68) | 0.35 (0.34) | -1.5 (0.90) ♦ | ? | -81 ◊ | 12 | 2 months |
|  | X | | | Plaque score * | Test | 0.22 (0.05) ♦ | 0.24 (0.15) ♦ | 0 (0.15) ♦ | No | 0 ◊ | 23 | 6 months |
|  |  |  |  |  | Control | 0.21 (0.05) ♦ | 0.22 (0.16) ♦ | -0.01 (0.16) ♦ | No | -5 ◊ | 24 | 6 months |
|  | XI | | | PI  Silness & Loë 1964 | Test | 2.15 (0.47) ♦ | 1.1 (0.39) ♦ | 1.08 (0.58) ♦ | Yes | -20 ◊ | 12 | 6 months |
|  |  |  |  |  | Control | 2.25 (0.28) ♦ | 1.19 (0.39) ♦ | 1.06 (0.44) ♦ | Yes | - 47 ◊ | 16 | 6 months |
|  | XIV | | | PI  Silness & Loë 1964 | Test | 1.87 (0.54) | 0.85 (0.33) | -1.17 (0.6) | Yes | -55 ◊ | 28 | 2 months |
|  |  |  |  |  | Control | 1.7 (0.50) | 1.5 (0.37) | -0.65 (0.46) | Yes | -12 ◊ | 22 | 2 months |
|  | XV | | | PI  Silness & Loë 1964 | Test | 0.88 (0.64) ♦ | 0.57 (0.79) ♦ | -0.31 ◊ | ? | -35 ◊ | 7 | 12 months |
|  |  |  |  |  | Control | 0.63 (0.52) ♦ | 0.86 (0.38) ♦ | +0.23 ◊ | ? | +37 ◊ | 7 | 12 months |
|  | | XIX | PI  ? | | Test | 1.0 (0.4) | 0.4 (0.3) | -0.6 ◊ | Yes | -60 ◊ | 23 | 6 months |
|  |  |  |  |  | Control | 0.9 (0.4) | 0.3 (0.2) | -0.6 ◊ | Yes | -67 ◊ | 26 | 6 months |
|  | | | | | | | | | | | | |
| **Percentage** | | I | Plaque score  * | | Test | 78.0 (15.1) | 35.4 (21.1) | -42.6 (16.4) ♦ | Yes | -55 ◊ | 39 | 12 months |
|  |  |  |  |  | Control | 78.9 (15.6) | 38.8 (24.2) | -40.0 (20.3) ♦ | Yes | -51 ◊ | 40 | 12 months |
|  |  | II | Plaque score  * | | Test | 71 (18) ♦ | 31 (21) ♦ | -41 (20) ♦ | Yes | -58 ◊ | 18 | 12 months |
|  |  |  |  |  | Control | 69 (19) ♦ | 27 (19) ♦ | -42 (25) ♦ | Yes | -61 ◊ | 17 | 12 months |
|  |  | III | Plaque score  * | | Test | 61.3 (19.8) | 35.9 (13.6) | -25.1 (23) ♦ | Yes | -41 ◊ | 15 | 12 months |
|  |  |  |  |  | Control | 62.7 (22.4) | 33.3 (13.2) | -28.3 (33.1) ♦ | Yes | -45 ◊ | 15 | 12 months |
|  |  | V | Plaque score * | | Test | 47.4 (28.2) ♦ | 40.5 (33.4) ♦ | -6.9 (38.7) ♦ | ? | -15 ◊ | 26 | 24 months |
|  |  |  |  |  | Control | 69.3 (29.2) ♦ | 40.2 (31.5) ♦ | -29.1 (35.7) ♦ | ? | -42 ◊ | 23 | 24 months |
|  |  | VI | Plaque score * | | Test | 59.7 (19) | 13.0 (1.6) | -46.7 (18.2) ♦ | Yes | -78 ◊ | 19 | 6 months |
|  |  |  |  |  | Control | 60.4 (27.1) | 12.1 (2.1) | -48.3 (28.4) ♦ | Yes | -80 ◊ | 20 | 6 months |
|  |  | VII | PI  (AinamoBay) | | Test | 23.8 (4.6) | 15.6 (3.1) | -8.3 (5.8) ♦ | No | -34 ◊ | 12 | 6 months |
|  |  |  |  |  | Control | 25.8 (7.1) | 19.4 (3.0) | -6.1 (8.3) ♦ | No | -25 ◊ | 12 | 6 months |
|  |  | IX | PI * | | Test | 79 (15.9) | 30.2 (23.7) | -48.3 (22.4) ♦ | Yes | -66 ◊ | 17 | 3 months |
|  |  |  |  |  | Control | 82.4 (13) | 30.1 (22.9) | -50.9 (15.9) ♦ | Yes | -63 ◊ | 17 | 3 months |
|  |  | XII | Plaque score  * | | Test | 37.61 (13.64) | 35.52 (16.42) ♦ | -2.09 ♦ | ? | -6 ◊ | 13 | 6 months |
|  |  |  |  |  | Control | 32.50 (11.68) | 33.11 (15.92) ♦ | +0.61 ♦ | ? | +2 ◊ | 12 | 6 months |
|  |  | XIII | Plaquescore  * | | Test | 73.7 (18.8) | 43.5 (16.9) | -30.62 (20.33) ♦ | Yes | -41 ◊ | 14 | 3 months |
|  |  |  |  |  | Control | 66.6 (22.9) | 42.6 (25.7) | -25.92 (23.50) ♦ | Yes | -36 ◊ | 15 | 3 months |
|  |  | XVII | Plaque score  * | | Test | 25.5 (12.03) ♦ | 21.1 (15.2) ♦ | -4.3 (12.03) ♦ | No | -17 ◊ | 20 | 6 months |
|  |  |  |  |  | Control | 19.9 (11.4) ♦ | 17.0 (9.9) ♦ | -2.9 (9.03) ♦ | No | -15 ◊ | 21 | 6 months |
|  |  | XX | PI  * | | Test | 70 (4) | 12 (10) | -58 ◊ | No | -83 ◊ | 8 | 24 months |
|  |  |  |  |  | Control | 68 (5) | 17 (12) | -51 ◊ | No | -75 ◊ | 8 | 24 months |

* Plaque scores: recorded as absence (0) and presence (1) of plaque on tooth surfaces

♦ = data obtained from the original author

◊ = data calculated by the author

**Appendix S57: Summary and overview of selected studies. Outcome data extraction with respect to parameters of interest.**

**Bleeding scores**

| **Value** | **ID# of selected studies** | **Index** | **Test/**  **Control**  **group** | **Mean**  **Baseline**  **scores** | **Mean**  **End**  **scores** | **Difference**  **(absolute)** | **Significant**  **(within group)** | **Difference**  **(relative %)** | **N** | **Evaluation**  **Period** |
| --- | --- | --- | --- | --- | --- | --- | --- | --- | --- | --- |
| **Mean** | IV | BOP | Test | 0.75 (0.44) ♦ | 0.23 (0.42) ♦ | -0.52 (0.56) ♦ | ? | -69 ◊ | 44 | 3 months |
|  |  |  | Control | 0.70 (0.46) ♦ | 0.31 (0.48) ♦ | -0.38 (0.59) ♦ | ? | -54 ◊ | 38 | 3 months |
|  | XVI | BOP | Test | 0.87 (0.21) | 0.15 (0.14) | -0.72 (0.27) ♦ | Yes | -83 ◊ | 10 | 6 months |
|  |  |  | Control | 0.78 (0.37) | 0.15 (0.25) | -0.23 (0.2) ♦ | Yes | -81 ◊ | 11 | 6 months |
|  | XIX | BOP | Test | 0.8 (0.2) | 0.2 (0.1) | -0.6 ◊ | Yes | -75 ◊ | 23 | 6 months |
|  |  |  | Control | 0.8 (0.2) | 0.4 (0.1) | -0.4 ◊ | Yes | -50 ◊ | 26 | 6 months |
|  | | | | | | | | | | |
| **Percentage** | I | BOP | Test | 63.7 (21.8) | 11.4 (18.9) | -52.4 (19.0) ♦ | Yes | -82 ◊ | 39 | 12 months |
|  |  |  | Control | 70.6 (23.8) | 23.5 (25.8) | -47.1 (25.4) ♦ | Yes | -67 ◊ | 40 | 12 months |
|  | II | BOP | Test | 86 (15) ♦ | 58 (18) ♦ | -27 (17) ♦ | Yes | -31 ◊ | 18 | 12 months |
|  |  |  | Control | 80 (20) ♦ | 60 (18) ♦ | -20 (21) ♦ | Yes | -25 ◊ | 17 | 12 months |
|  | III | BOP | Test | 77.7 (19.7) | 10.0 (7.1) | -65.2 (24.4) ♦ | Yes | -84 ◊ | 15 | 12 months |
|  |  |  | Control | 63.8 (21.3) | 13.7 (10.3) | -49.6 (25.7) ♦ | Yes | -78 ◊ | 15 | 12 months |
|  | V | BOP | Test | 70.4 (15.3) ♦ | 34.1 (26.6) ♦ | -36.3 (33.8) ♦ | ? | - 52 ◊ | 26 | 24 months |
|  |  |  | Control | 82.7 (15.3) ♦ | 47.1 (21.4) ♦ | -35.6 (27.7) ♦ | ? | -43 ◊ | 23 | 24 months |
|  | VI | BOP | Test | 61.5 (17.7) | 9.4 (0.6) | -52.1 (17.5) ♦ | Yes | -85 ◊ | 19 | 6 months |
|  |  |  | Control | 56.2 (18.2) | 15.5 (3.8) | -40.7 (16.8) ♦ | Yes | -72 ◊ | 20 | 6 months |
|  | VII | BOP  Muhlemann&Son | Test | 39.3 (6.5) | 10.3 (2.0) | -29 (6.9) ♦ | Yes | -74 ◊ | 12 | 6 months |
|  |  |  | Control | 34.3 (6.1) | 11.0 (3.0) | -23.3 (6.8) ♦ | Yes | -68 ◊ | 12 | 6 months |
|  | VIII | BOP  Ainamo | Test | 93.57 (0.11) | 25.21 (0.13) | -68.34 (0.13) ♦ | Yes | -73 ◊ | 14 | 2 months |
|  |  |  | Control | 95.0 (0.10) | 37.7 (0.12) | -57.3 (0.20) ♦ | Yes | -60 ◊ | 12 | 2 months |
|  | IX | BOP | Test | 72.9 (17.2) | 31.5 (25.4) | -41.4 (21.0) ♦ | Yes | -57 ◊ | 17 | 3 months |
|  |  |  | Control | 70.9 (22.4) | 32.8 (25.1) | -38.0 (26.1) ♦ | Yes | -54 ◊ | 17 | 3 months |

|  | X | BOP | Test | 64.2 (10) ♦ | 16 (8) ♦ | -49 (14) ♦ | ? | -76 ◊ | 23 | 6 months |
| --- | --- | --- | --- | --- | --- | --- | --- | --- | --- | --- |
|  |  |  | Control | 63 (13) ♦ | 19 (7) ♦ | - 46 (15) ♦ | ? | -73 ◊ | 24 | 6 months |
|  | XII | BOP  Mühlemann & Son 1971 | Test | 26.80 (12.36) | 5.49 (3.61) ♦ | -21.31 (11.2) ♦ | Yes | -80 ◊ | 13 | 6 months |
|  |  |  | Control | 24.36 (11.25) | 11.06 (3.82) ♦ | -13.3 (9.5) ♦ | Yes | -55 ◊ | 12 | 6 months |
|  | XIII | BOP | Test | 75.8 (22.9) | 56.3 (16.5) | -18.03 (17.49) ♦ | Yes | -26 ◊ | 14 | 3 months |
|  |  |  | Control | 67.0 (18.2) | 65.3 (17.4) | -2.3 (20.71) ♦ | No | -3 ◊ | 15 | 3 months |
|  | XIV | BOP | Test | 96 (10.98) | 32.23 (17.82) | -63.8 (19.5) | Yes | -67 ◊ | 28 | 2 months |
|  |  |  | Control | 93.1 (12.48) | 38.5 (13.07) | -50.6 (22.3) | Yes | -59 ◊ | 22 | 2 months |
|  | XV | BOP | Test | 88 (35) ♦ | 43 (53) ♦ | -45 ◊ | ? | -51 ◊ | 7 | 12 months |
|  |  |  | Control | 100 (0) ♦ | 71 (49) ♦ | -29 ◊ | ? | -29 ◊ | 7 | 12 months |
|  | XVII | BOP | Test | 60.7 (17.8) ♦ | 29.7 (9.9) ♦ | -31.0 (11.8) ♦ | Yes | -51 ◊ | 20 | 6 months |
|  |  |  | Control | 51.6 (19.2) ♦ | 33.9 (14.4) ♦ | -17.7 (18.3) ♦ | Yes | -34 ◊ | 21 | 6 months |
|  | XVIII | BOP | Test | 44.3 (17.7) ♦ | 34.8 (21.0) ♦ | -9.6 (25.9) ♦ | ? | -22 ◊ | 18 | 24 months |
|  |  |  | Control | 63.4 (17.8) ♦ | 55.4 (28.9) ♦ | -8.0 (29.2) ♦ | ? | -13 ◊ | 17 | 24 months |
|  | XX | BOP | Test | 72 (15) | 14 (5) | -58 ◊ | Yes | -81 ◊ | 8 | 24 months |
|  |  |  | Control | 71 (8) | 22 (16) | -49 ◊ | Yes | -69 ◊ | 8 | 24 months |

**Appendix S58: Summary and overview of selected studies. Outcome data extraction with respect to parameters of interest.**

**Mean probing pockets depth**

| **Value** | **ID# of selected studies** | **Test/**  **Control**  **group** | **Mean**  **Baseline**  **scores** | **Mean**  **End**  **scores** | **Difference**  **(absolute)** | **Significant**  **(within group)** | **Difference**  **(relative %)** | **N** | **Evaluation period** |
| --- | --- | --- | --- | --- | --- | --- | --- | --- | --- |
| **Mean** | I | Test | 3.88 (0.98) | 2.54 (0.40) | -1.34 (0.77) ♦ | Yes | -35 ◊ | 39 | 12 months |
|  |  | Control | 3.84 (0.73) | 3.05 (0.59) | -0.77 (0.50) ♦ | Yes | -20 ◊ | 40 | 12 months |
|  | II | Test | 4.3 (0.8) ♦ | 3.0 (0.40) ♦ | -1.3 (0.6) ♦ | Yes | -30 ◊ | 18 | 12 months |
|  |  | Control | 4.1 (0.7) ♦ | 3.0 (0.5) ♦ | -1.0 (0.7) ♦ | Yes | -24 ◊ | 17 | 12 months |
|  | III | Test | 4.27 (0.71) | 2.64 (0.42) | -1.61 (0.60) | Yes | -38 ◊ | 15 | 12 months |
|  |  | Control | 4.09 (0.62) | 3.17 (0.46) | -0.92 (0.47) | Yes | -22 ◊ | 15 | 12 months |
|  | VI | Test | 4.3 (1.1) | 2.7 (0.6) | -1.6 (0.5) | Yes | -37 ◊ | 19 | 6 months |
|  |  | Control | 4.5 (1.1) | 3.3 (0.8) | -1.2 (0.3) | Yes | -27 ◊ | 20 | 6 months |
|  | VIII | Test | 4.86 (0.74) | 3.37 (0.39) | -1.49 (0.71) ♦ | Yes | -31 ◊ | 14 | 2 months |
|  |  | Control | 4.93 (0.31) | 4.21 (0.19) | -0.72 (0.25) ♦ | Yes | -15 ◊ | 12 | 2 months |
|  | IX | Test | 3.8 (0.5) | 2.7 (0.4) | -1.08 (0.40) | Yes | -28 ◊ | 17 | 3 months |
|  |  | Control | 3.6 (0.5) | 3.0 (0.6) | -0.63 (0.26) | Yes | -18 ◊ | 17 | 3 months |
|  | XI | Test | 4.04 (0.57) ♦ | 2.58 (0.37) ♦ | -1.46 (0.16) ♦ | Yes | -36 ◊ | 12 | 6 months |
|  |  | Control | 3.7 (0.69) ♦ | 2.47 (0.49) ♦ | -1.22 (0.63) ♦ | Yes | -33 ◊ | 16 | 6 months |
|  | XIII | Test | 4.0 (0.7) | 3.0 (0.8) | -1.0 (0.35) ♦ | Yes | -25 ◊ | 14 | 3 months |
|  |  | Control | 3.9 (0.6) | 3.3 (0.5) | -0.6 (0.51) ♦ | Yes | -15 ◊ | 15 | 3 months |
|  | XVI | Test | 4.63 (0.97) | 3.12 (0.71) | -1.39 (0.74) ♦ | Yes | -33 ◊ | 10 | 6 months |
|  |  | Control | 4.21 (0.74) | 3.52 (0.76) | -0.66 (0.69) ♦ | Yes | -16 ◊ | 11 | 6 months |
|  | XVII | Test | 4.1 (0.97) ♦ | 2.9 (0.6) ♦ | -1.2 (0.5) ♦ | Yes | -29 ◊ | 20 | 6 months |
|  |  | Control | 4.1 (0.9) ♦ | 3.3 (0.8) ♦ | -0.7 (0.6) ♦ | Yes | -17 ◊ | 21 | 6 months |
|  | XIX | Test | 4.4 (0.6) | 3.0 (0.4) | -1.4 ◊ | Yes | -32 ◊ | 23 | 6 months |
|  |  | Control | 4.4 (0.5) | 3.4 (0.5) | -1.0 ◊ | Yes | -23 ◊ | 26 | 6 months |

|  | XX | Test | 4.8 (0.7) | 2.7 (0.2) | | -2.1 ◊ | Yes | -44 ◊ | 8 | 24 months |
| --- | --- | --- | --- | --- | --- | --- | --- | --- | --- | --- |
|  |  | Control | 4.5 (0.8) | 2.9 (0.6) | | -1.6 ◊ | Yes | -36 ◊ | 8 | 24 months |
|  | | | | | | | | | | |
| **PD**  **> 4mm** | IV | Test | 4.53 (1.79) ♦ | 2.86 (0.81) ♦ | -1.65 (1.59) ♦ | | ? | -36 ◊ | 44 | 3 months |
|  |  | Control | 4.47 (1.60) ♦ | 3.24 (1.11) ♦ | -1.23 (1.35) ♦ | | ? | -28 ◊ | 38 | 3 months |
|  | V | Test | 6.01 (0.47) ♦ | 3.95 (0.77) ♦ | -2.06 (0.83) ♦ | | Yes | -34 ◊ | 26 | 24 months |
|  |  | Control | 6.17 (0.80) ♦ | 4.45 (0.81) ♦ | -1.72 (0.96) ♦ | | ? | -28 ◊ | 23 | 24 months |
|  | X | Test | 4.35 (0.37) ♦ | 2.98 (0.21) ♦ | -1.6 (0.96) ♦ | | Yes | -37 ◊ | 23 | 6 months |
|  |  | Control | 4.39 (0.45) ♦ | 3.15 (0.27) ♦ | -1.49 (0.99) ♦ | | Yes | -34 ◊ | 24 | 6 months |
|  | XII | Test | 6.4 (0.43) | 3.12 (0.37) | -3.28 (0.41) | | ? | -51 ◊ | 13 | 6 months |
|  |  | Control | 6.17 (0.49) | 3.72 (0.41) | -2.45 (0.50) | | ? | -40 ◊ | 12 | 6 months |
|  | XIV | Test | 5.79 (0.61) | 3.59 (0.72) | -2.19 (0.67) | | Yes | -38 ◊ | 28 | 2 months |
|  |  | Control | 5.62 (0.55) | 4.11 (0.54) | -1.51 (0.45) | | Yes | -27 ◊ | 22 | 2 months |

|  | XV | Test | 7.13 (2.10) ♦ | 4.86 (1.77) ♦ | -2.27 ◊ | | ? | -32 ◊ | 7 | 12 months |
| --- | --- | --- | --- | --- | --- | --- | --- | --- | --- | --- |
|  |  | Control | 6.75 (1.39) ♦ | 4.86 (1.07) **♦** | -1.89 ◊ | | ? | -28 ◊ | 7 | 12 months |
|  | | | | | | | | | | |
| **PPD**  **4-6mm** | I | Test | 4.76 (0.26) ♦ | 2.82 (0.41) ♦ | -1.9 (0.5) | | ? | -40 ◊ | 39 | 12 months |
|  |  | Control | 4.74 (0.20) ♦ | 3.45 (0.56) ♦ | -1.3 (0.5) | | ? | -27 ◊ | 40 | 12 months |
|  | II | Test | 5.1 (0.2) ♦ | 3.2 (0.4) ♦ | -1.9 (0.4) ♦ | | Yes | -37 ◊ | 18 | 12 months |
|  |  | Control | 5.0 (0.2) ♦ | 3.4 (0.7) ♦ | -1.5 (0.7) ♦ | | Yes | -30 ◊ | 17 | 12 months |
|  | III | Test | 4.89 (0.14) ♦ | 2.58 (0.31) ♦ | -2.30 (0.29) | | ? | -47 ◊ | 15 | 12 months |
|  |  | Control | 4.74 (0.15) ♦ | 3.33 (0.41) ♦ | -1.41 (0.47) | | ? | -30 ◊ | 15 | 12 months |
|  | XVII | Test | 5.02 (0.2) ♦ | 3.5 (0.4) ♦ | -1.5 (0.4) ♦ | | Yes | -30 ◊ | 20 | 6 months |
|  |  | Control | 5.0 (0.2) ♦ | 3.9 (0.5) ♦ | -1.0 (0.4) ♦ | | Yes | -20 ◊ | 21 | 6 months |
|  | XIX | Test | ? | ? | -1.72 (0.42) | | ? | ? | 23 | 6 months |
|  |  | Control | ? | ? | -1.37 (0.36) | | ? | ? | 26 | 6 months |
|  | | | | | | | | | | |
| **PD**  **≥ 6 mm** | I | Test | 7.72 (0.64) **♦** | 3.63 (0.65) **♦** | | -4.1 (0.7) | ? | -53 ◊ | 39 | 12 months |
|  |  | Control | 7.84 (0.64) **♦** | 4.75 (1.45) **♦** | | -3.1 (1.4) | ? | -40 ◊ | 40 | 12 months |
|  | II | Test | 7.8 (2.2) **♦** | 3.9 (1.2) **♦** | | -3.9 (1.4) **♦** | Yes | -50 ◊ | 18 | 12 months |
|  |  | Control | 8.0 (0.8) **♦** | 4.9 (1.6) **♦** | | -3.2 (1.4) **♦** | Yes | -40 ◊ | 17 | 12 months |
|  | III | Test | 8.18 (0.97) ♦ | 3.94 (1.06) ♦ | | -4.18 (1.42) | ? | -51 ◊ | 15 | 12 months |
|  |  | Cotnrol | 8.22 (1.44) ♦ | 5.43 (1.42) ♦ | | -2.78 (1.09) | ? | -34 ◊ | 15 | 12 months |
|  | VI | Test | 6.9 (0.7) | 3.8 (0.8) | | -3.1 (0.6) | Yes | -45 ◊ | 19 | 6 months |
|  |  | Control | 7.1 (0.6) | 4.7 (0.8) | | -2.4 (0.7) | Yes | -34 ◊ | 20 | 6 months |
|  | VII | Test | 7.73 (0.9) | 3.6 (1.38) | | -4.13 (1.44) | Yes | -53 ◊ | 12 | 6 months |
|  |  | Control | 7.54 (0.57) | 4.08 (0.90) | | -3.28 (0.60) | Yes | -44 ◊ | 12 | 6 months |
|  | XII | Test | 5.47 (0.22) ♦ | 1.59 (0.17) ♦ | | -3.88 (0.93) | ? | -71 ◊ | 13 | 6 months |
|  |  | Control | 5.55 (0.30) ♦ | 2.1 (0.15) ♦ | | -3.45 (0.89) | ? | -62 ◊ | 12 | 6 months |

|  | XVII | Test | 7.7 (0.5) ♦ | 4.6 (0.7) ♦ | -3.1 (0.8) ♦ | Yes | -40 ◊ | 20 | 6 months |
| --- | --- | --- | --- | --- | --- | --- | --- | --- | --- |
|  |  | Control | 7.7 (0.8) ♦ | 5.9 (1.5) ♦ | -1.8 (1.1) ♦ | Yes | -23 ◊ | 21 | 6 months |
|  | XIX | Test | ? | ? | -3.18 (0.92) | ? | ? | 23 | 6 months |
|  |  | Control | ? | ? | -2.46 (0.89) | ? | ? | 26 | 6 months |

**Appendix S59: Summary and overview of the selected studies. Outcome data extraction with respect to parameters of interest**

**Mean clinical attachment level**

| **Value** | **ID# of selected studies** | **Index** | **Test/**  **Control**  **group** | **Mean**  **Baseline**  **scores** | **Mean**  **End**  **scores** | **Difference**  **(absolute)** | **Significant**  **(within group)** | **Difference**  **(relative %)** | **N** | **Evaluation**  **Period** |
| --- | --- | --- | --- | --- | --- | --- | --- | --- | --- | --- |
| **Mean** | I | CAL | Test | 4.33 (1.08) | 3.23 (0.74) | +1.10 (0.63) ♦ | Yes | +25 ◊ | 39 | 12 months |
|  |  |  | Control | 4.32 (0.97) | 3.59 (0.89) | +0.71 (0.45) ♦ | Yes | +16 ◊ | 40 | 12 months |
|  | II | CAL | Test | 4.9 (1.3) ♦ | 3.8 (1.1) ♦ | +1.1 (0.6) ♦ | Yes | +22 ◊ | 18 | 12 months |
|  |  |  | Control | 4.4 (1.1) ♦ | 3.6 (1.0) ♦ | +0.8 (0.6) ♦ | Yes | +12 ◊ | 17 | 12 months |
|  | III | CAL | Test | 4.47 (0.84) | 3.32 (0.74) | +1.61 (0.60) | Yes | +36 ◊ | 15 | 12 months |
|  |  |  | Control | 4.23 (0.50) | 3.63 (0.58) | +0.93 (0.47) | Yes | +22 ◊ | 15 | 12 months |
|  | VI | CAL | Test | 4.7 (1.1) | 3.3 (0.6) | +1.4 (0.6) | Yes | +30 ◊ | 19 | 6 months |
|  |  |  | Control | 5.0 (1.2) | 4.0 (1.0) | +1.0 (0.3) | Yes | +20 ◊ | 20 | 6 months |
|  | VIII | CAL | Test | 5.28 (0.81) | 3.91 (0.51) | +1.37 (0.69) ♦ | Yes | +26 ◊ | 14 | 2 months |
|  |  |  | Control | 5.49 (0.45) | 4.70 (0.61) | +0.78 (0.62) ♦ | Yes | +14 ◊ | 12 | 2 months |
|  | IX | CAL | Test | 4.4 (0.6) | 3.6 (0.7) | +0.76 (0.42) | Yes | +17 ◊ | 17 | 3 months |
|  |  |  | Control | 4.2 (0.7) | 3.7 (0.7) | +0.53 (0.27) | Yes | +13 ◊ | 17 | 3 months |
|  | XI | CAL | Test | 3.83 (1.05) ♦ | 2.84 (1.24) ♦ | +0.99 (0.90) ♦ | Yes | +26 ◊ | 12 | 6 months |
|  |  |  | Control | 3.34 (1.38) ♦ | 2.43 (1.11) ♦ | +0.91 (0.55) ♦ | Yes | +27 ◊ | 16 | 6 months |
|  | XIII | CAL | Test | 4.8 (0.8) | 3.9 (0.8) | +0.84 (0.47) ♦ | Yes | +19 ◊ | 14 | 3 months |
|  |  |  | Control | 4.7 (1.2) | 4.2 (1.1) | +0.48 (0.65) ♦ | Yes | +11 ◊ | 15 | 3 months |
|  | XVI | PAL | Test | 4.97 (1.01) | 4.05 (1.34) | +0.92 (0.84) ♦ | Yes | +19 ◊ | 10 | 6 months |
|  |  |  | Control | 4.55 (0.72) | 4.07 (0.59) | +0.48 (0.53) ♦ | Yes | +11 ◊ | 11 | 6 months |
|  | XVII | CAL | Test | 4.7 (1.2) ♦ | 3.9 (1.02) ♦ | +0.8 (0.3) ♦ | Yes | +17◊ | 20 | 6 months |
|  |  |  | Control | 4.7 (1.5) ♦ | 4.2 (1.5) ♦ | +0.5 (0.5) ♦ | Yes | +11 ◊ | 21 | 6 months |
|  | XIX | CAL | Test | 3.9 (1.1) | 3.2 (1.0) | +0.7 ◊ | No | +18 ◊ | 23 | 6 months |
|  |  |  | Control | 4.0 (1.3) | 3.6 (1.1) | +0.4 ◊ | No | +10 ◊ | 26 | 6 months |

|  | XX | PAL | Test | ? | ? | +1.1 (0.3) | Yes | ? | 8 | 24 months |
| --- | --- | --- | --- | --- | --- | --- | --- | --- | --- | --- |
|  |  |  | Control | ? | ? | +0.8 (0.4) | Yes | ? | 8 | 24 months |
|  | | | | | | | | | | |
| **CAL**  **Baseline PPD > 4mm** | IV | CAL | Test | 5.47 (2.07) ♦ | 4.23 (1.61) ♦ | +1.23 (1.66) ♦ | ? | +23 ◊ | 44 | 3months |
|  |  |  | Control | 5.41 (1.96) ♦ | 4.55 (1.68) ♦ | +0.86 (1.46) ♦ | ? | +16 ◊ | 38 | 3 months |
|  | V | CAL | Test | 6.35 (1.40 ) ♦ | 5.06 (1.54) ♦ | +1.29 (0.83) ♦ | ? | +20 ◊ | 26 | 24 months |
|  |  |  | Control | 5.84 (1.52) ♦ | 4.99 (1.23) ♦ | +0.84 (0.91) ♦ | ? | +14 ◊ | 23 | 24 months |
|  | X | CAL | Test | 5.52 (0.69) ♦ | 4.61 (0.84) ♦ | +0.9 (0.38) ♦ | No | +16 ◊ | 23 | 6 months |
|  |  |  | Control | 5.32 (0.70) ♦ | 4.44 (0.65) ♦ | +0.89 (0.40) ♦ | No | +17 ◊ | 24 | 6 months |
|  | XII | RAL | Test | 8.15 (0.55) | 6.27 (0.89) ♦ | +1.88 (0.21) ♦ | ? | +23 ◊ | 13 | 6 months |
|  |  |  | Control | 8.22 (1.02) | 6.59 (0.85) ♦ | 1.63 (0.27) ♦ | ? | +20 ◊ | 12 | 6 months |
|  | XIV | CAL | Test | 5.8 (1.11) | 3.87 (0.99) | +1.92 (0.67) | Yes | +33 ◊ | 28 | 2 months |
|  |  |  | Control | 5.34 (0.72) | 4.17 (0.85) | +1.15 (0.46) | Yes | +22 ◊ | 22 | 2 months |

|  | XV | CAL | Test | 8.0 (2.1) | ? | ? | ? | ? | 7 | 12 months |
| --- | --- | --- | --- | --- | --- | --- | --- | --- | --- | --- |
|  |  |  | Control | 7.3 (1.9) | ? | ? | ? | ? | 7 | 12 months |
|  | | | | | | | | | | |
| **CAL**  **Baseline PPD 4-6mm** | I | CAL | Test | 5.17 (0.62) ♦ | 3.56 (0.76) ♦ | +1.6 (0.4) | ? | +31 ◊ | 39 | 12 months |
|  |  |  | Control | 5.16 (0.61) ♦ | 3.97 (0.88) ♦ | +1.2 (0.5) | ? | +23 ◊ | 40 | 12 months |
|  | II | CAL | Test | 5.8 (1.0) ♦ | 4 (1.0) ♦ | +1.7 (0.6) ♦ | Yes | + 29 ◊ | 18 | 12 months |
|  |  |  | Control | 5.3 (0.9) ♦ | 4.1 (1.1) ♦ | +1.4 (0.5) ♦ | Yes | + 26 ◊ | 17 | 12 months |
|  | III | CAL | Test | 5.19 (0.49) ♦ | 3.25 (0.56) ♦ | +1.96 (0.44) | ? | + 38 ◊ | 15 | 12 months |
|  |  |  | Control | 4.87 (0.47) ♦ | 3.76 (0.50) ♦ | +1.11 (0.63) | ? | + 23 ◊ | 15 | 12 months |
|  | XVII | CAL | Test | 5.7 (0.8) ♦ | 4.4 (1.02) ♦ | +1.3 (0.3) ♦ | Yes | +23 ◊ | 20 | 6 months |
|  |  |  | Control | 5.7 (0.98) ♦ | 4.8 (1.3) ♦ | +0.8 (0.6) ♦ | Yes | +14 ◊ | 21 | 6 months |

|  | XVIII | PAG | Test | ? | ₪ | ? | No | ? | 18 | 24 months |
| --- | --- | --- | --- | --- | --- | --- | --- | --- | --- | --- |
|  |  |  | Control | ? | ₪ | ? | ? | ? | 17 | 24 months |
|  | XIX |  | Test | ? | ? | +0.88 (0.38) | ? | ? | 23 | 6 months |
|  |  |  | Control | ? | ? | +0.68 (0.44) | ? | ? | 26 | 6 months |

|  | | | | | | | | | | |
| --- | --- | --- | --- | --- | --- | --- | --- | --- | --- | --- |
| **CAL**  **Baseline PPD ≥ 6 mm** | I | CAL | Test | 8.06 (1.05) ♦ | 4.70 (0.89) ♦ | +3.4 (0.8) | ? | +42 ◊ | 39 | 12 months |
|  |  |  | Control | 8.18 (1.20) ♦ | 5.61 (1.88) ♦ | +2.6 (1.2) | ? | +32 ◊ | 40 | 12 months |
|  | II | CAL | Test | 8.5 (2.6) ♦ | 5.3 (2.3) ♦ | +3.2 (1.3) ♦ | Yes | +38 ◊ | 18 | 12 months |
|  |  |  | Control | 8.4 (1.7) ♦ | 5.7 (2.1) ♦ | +2.7 (1.3) ♦ | Yes | +32 ◊ | 17 | 12 months |
|  | III | CAL | Test | 8.47 (1.16) ♦ | 5.04 (1.21) ♦ | +3.35 (1.23) | ? | +40 ◊ | 15 | 12 months |
|  |  |  | Control | 8.35 (1.66) ♦ | 6.01 (1.62) ♦ | +2.32 (1.11) | ? | +28 ◊ | 15 | 12 months |
|  | VI | CAL | Test | 7.7 (0.7) | 4.7 (0.8) | +3.0 (0.7) | Yes | -39 ◊ | 19 | 6 months |
|  |  |  | Control | 7.8 (0.6) | 5.8 (1.0) | +2.0 (0.8) | Yes | -26 ◊ | 20 | 6 months |
|  | VII | CAL | Test | 9.93 (1.6) | 7.70 (2.60) | +2.34 (1.57) | Yes | +24 ◊ | 12 | 6 months |
|  |  |  | Control | 10.00 (1.29) | 7.50 (1.17) | +2.49 (1.02) | Yes | +25 ◊ | 12 | 6 months |
|  | XII | CAL | Test | 7.34 (0.42) ♦ | 5.12 (0.48) ♦ | +2.22(1.18) | ? | +30 ◊ | 13 | 6 months |
|  |  |  | Control | 7.61 (0.94) ♦ | 5.21 (0.23) ♦ | +2.40 (0.57) | ? | +32 ◊ | 12 | 6 months |

|  | XVII | CAL | Test | 8.1 (0.7) ♦ | 5.8 (0.9) ♦ | +2.3 (0.5) ♦ | Yes | +28 ◊ | 20 | 6 months |
| --- | --- | --- | --- | --- | --- | --- | --- | --- | --- | --- |
|  |  |  | Control | 8.2 (1.5) ♦ | 6.8 (1.8) ♦ | +1.3 (0.6) ♦ | Yes | +16 ◊ | 21 | 6 months |
|  | XVIII | PAG | Test | ? | ₪ | +1.7 (0.3) | No | ? | 18 | 24 months |
|  |  |  | Control | ? | ₪ | +0.3 (0.3) | ? | ? | 17 | 24 months |
|  | XIX | CAL | Test | ? | ? | +1.97 (0.79) | ? | ? | 23 | 6 months |
|  |  |  | Control | ? | ? | + 1.46 (0.70) | ? | ? | 26 | 6 months |

*** If change in CAL scores are depicted as positive there is gain of attachment**

**RAL= relative attachment level**

**PAG= Probing attachment gain**

**Appendix S60: Limitations of this systematic review**

1. The selected papers are quite heterogeneous and many additional factors could have influenced the outcomes. As examples, drug dosage and plaque control are important factors that should be taken in consideration (Herrera et al. 2008). Trial design, length of follow-up, disease entity, disease severity, and disease activity of the patient populations under investigation differ considerably among most studies. Furthermore, the heterogeneity regarding the antibiotics daily dosage and length of drug regimens makes definitive conclusions about use in clinical practice difficult (Kaner et al. 2007).
2. The quality of debridement may also influence the results. Very few studies have reported on the thoroughness of SRP, the time employed, who the operator was, whether it was done with or without local anesthesia and the time between treatment sessions. All these factors may account for the variability of the magnitude of changes observed some studies report high levels of plaque at the end of the study (Herrera et al. 2002).
3. Not all of the included studies used smoking as an exclusion criterion (see Appendix S6 for details). It is well known that smoking can reduce the response to non-surgical periodontal therapy (Ah et al. 1994, McGuire & Nunn, 1996). However, the smoking habits of the included participants was not frequently described, neither was the influence of the smoking habits on the treatment effect evaluated by most. Therefore, a meta-analysis on this part could not be assessed. Mainly because smokers were excluded in a large extent of the included studies it is reasonable to hypothesize that the confounding effect of smoking status was mitigated on the results of the meta-analysis. (Sgolastra et al. 2012). When the adjunctive use of amx+met was evaluated in a RCT between smokers and non-smokers for initial treatment of chronic periodontitis, both groups improved regarding clinical parameters. Although smokers with chronic periodontitis benefited less than non-smokers from the treatment by the combination of SRP+amx+met (Faveri et al. 2014).
4. The short-term evaluation of the included studies deserves a cautionary note. Indeed, longitudinal monitoring of these subjects will be important in order to determine whether this combination of therapies would produce sustained beneficial changes in the subgingival microbial profile and periodontal clinical parameters over time. Nevertheless, it has been suggested that the short-term changes in the microbial profile may determine long-term periodontal clinical stability (Mestnik et al. 2010).
5. Although no evidence of publication bias was observed, possibility of publication bias, cannot be excluded, because only a small number of studies (± 10) were included in the meta-analysis and funnel plots (Sgolastra et al. 2012).
6. Even if the difference of means (DiffM) was used to pool the data, none of the included studies reported a proven normal distribution of data. Therefore, the normal distribution of data was only supposed. Consequently, this issue should be considered when interpreting the results of the present meta-analysis (Sgolastra et al. 2012).
7. The few studies that have diagnosed patients based on the presence of a specific marker pathogen may have bias the generalizability of the outcome (see Appendix S5 for details). The impact of this could not be assessed because no information was provided on how many patients were excluded to meet microbiological entry criteria. Also Guerrero et al. (2014) observed that the effect of the adjunctive antimicrobials was not modified by the baseline microbiological status in the primary analysis.
8. However, when considering prescribing antibiotic use one needs to be concerned about several issues. These include antibiotic: allergic reactions, resistance, changes or re-equilibration of the oral (and gastrointestinal) microbiome, and the emergence of resistant bacterial strains. Assessments concerning allergic reactions vary, but data suggests a prevalence of approximately 5% for amoxicillin (Bigby et al. 1986). Furthermore treating periodontitis with amx+met facilitates oral recolonization by a health-compatible oral microbiome (Matarazzo et al. 2008, Silva et al. 2011). In addition resistance to metronidazole has rarely been reported (Soares et al. 2012) and short courses of combination therapy with two antibiotics prevents emergence of resistant strains (D'Agata et al. 2008).

**Appendix S61: Additional references included in the Appendices and not provided in the main document**

Ah MK, Johnson GK, Kaldahl WB, Patil KD, Kalkwarf KL. The effect of smoking on the response to periodontal therapy. *Journal of Clinical Periodontology* 1994; **21**: 91-97.

Ainamo J, Bay I. Problems and proposals for recording gingivitis and plaque. *International Dental Journal* 1975; **25:** 229-235.

Akincibay H, Orsal SO, Sengün D, Tözüm TF. Systemic administration of doxycycline versus metronidazole plus amoxicillin in the treatment of localized aggressive periodontitis: a clinical and microbiologic study. *Quintessence International* 2008; **39:** 33-39.

Baldan N, Freeman E. Antimicrobials in periodontitis: a clinical approach. *University of Toronto Dental Journal* 1991; **4**: 14-16.

Beliveau D, Magnusson I, Bidwell JA, Zapert EF, Aukhil I, Wallet SM, Shaddox LM. Benefits of early systemic antibiotics in localized aggressive periodontitis: a retrospective study. *Journal of Clinical Periodontology* 2012; **39:** 1075-1081.

Bigby M, Jick S, Jick H, Arndt K. Drug-induced cutaneous reactions. A report

from the Boston Collaborative Drug Surveillance Program on 15,438 consecutive

inpatients, 1975 to 1982. *Journal American Medical Association* 1986; **256**: 3358-3363.

Bonito AJ, Lohr KN, Lux L, Sutton S, Jackman A, Whitener L, Evensen C. Effectiveness of antimicrobial adjuncts to scaling and root-planing therapy for periodontitis. *Evidence report technology assessment (Summ)* 2004; **88:** 1-4.

Buchmann R, Müller RF, Heinecke A, Lange DE. *Actinobacillus actinomycetemcomitans* in destructive periodontal disease. Three-year follow-up results. *Journal of Periodontology* 2000; **71:** 444-453.

Ciancio S. Use and abuse of antibiotics in periodontal therapy. *Dental Economics* 1993; **83**: 98 100.

D'Agata EM, Dupont-Rouzeyrol M, Magal P, Olivier D, Ruan S. The impact of different antibiotic regimens on the emergence of antimicrobial-resistant bacteria. *PLoS One* 2008; 3:e4036.

De Graaff J, van Winkelhoff AJ, Goené RJ. The role of *Actinobacillus actinomycetemcomitans* in periodontal disease. *Infection* 1989; **17:** 269-271.

Egger M, Smith GD, Sterne JA. Uses and abuses of meta-analysis. *Clinical Medicine* 2001; **1:** 478-484.

Faveri M, Rebello A, de Oliveira Dias R, Borges-Junior I, Duarte PM, Figueiredo LC, Feres M. Clinical and microbiologic effects of adjunctive metronidazole plus amoxicillin in the treatment of generalized chronic periodontitis: smokers versus non-smokers. *Journal of Periodontology* 2014; **85:** 581-591.

Gaggl AJ, Rainer H, Grund E, Chiari FM. Local oxygen therapy for treating acute necrotizing periodontal disease in smokers. *Journal of Periodontology* 2006; **77:** 31-38.

Johnson JD, Chen R, Lenton PA, Zhang G, Hinrichs JE, Rudney JD. Persistence of extracrevicular bacterial reservoirs after treatment of aggressive periodontitis. *Journal of Periodontology* 2008; **79:** 2305-2312.

López NJ, Gamonal JA, Martinez B. Repeated metronidazole and amoxicillin treatment of periodontitis. A follow-up study. *Journal of Periodontology* 2000; **71:** 79-89.

Machtei EE, Younis MN. The use of 2 antibiotic regimens in aggressive periodontitis: comparison of changes in clinical parameters and gingival crevicular fluid biomarkers. *Quintessence International* 2008; **39:** 811-819.

McGuire MK, Nunn ME. Prognosis versus actual outcome. III. The effectiveness of clinical parameters in accurately predicting tooth survival. *Journal of Periodontology* 1996; **67:** 666-674.

Moreira RM, Feres-Filho EJ. Comparison between full-mouth scaling and root planing and quadrant-wise basic therapy of aggressive periodontitis: 6-month clinical results. *Journal of Periodontology* 2007; **78:** 1683-1688.

Mühlemann HR, Son S. Gingival sulcus bleeding--a leading symptom in initial gingivitis. *Helvetica odontologica acta* 1971; **15:** 107-113.

Müller HP, Heinecke A, Borneff M, Kiencke C, Knopf A, Pohl S. Eradication of *Actinobacillus actinomycetemcomitans* from the oral cavity in adult periodontitis. *Journal of Periodontal Research* 1998; **33:** 49-58.

Pahkla ER, Koppel T, Naaber P, Saag M, Loivukene K. The efficacy of non-surgical and systemic antibiotic treatment on smoking and non-smoking periodontitis patients. *Stomatologija* 2006; **8:** 116-121.

Pavicić MJ, van Winkelhoff AJ, Douqué NH, Steures RW, de Graaff J. Microbiological and clinical effects of metronidazole and amoxicillin in *Actinobacillus actinomycetemcomitans*-associated periodontitis. A 2-year evaluation. *Journal of Clinical Periodontol*ogy 1994; **21:** 107-112.

Rodrigues AS, Lourenção DS, Lima Neto LG, Pannuti CM, Hirata RD, Hirata MH, Lotufo RF, De Micheli G. Clinical and microbiologic evaluation, by real-time polymerase chain reaction, of non-surgical treatment of aggressive periodontitis associated with amoxicillin and metronidazole. *Journal of Periodontology* 2012; **83:** 744-752.

Silness J, Löe H. Periodontal disease in pregnancy. II. Correlation between oral hygiene and periodontal disease. *Acta odontologica Scandinavica* 1964; **22:** 121-135.

Tinoco EM, Beldi MI, Campedelli F, Lana M, Loureiro CA, Bellini HT, Rams TE, Tinoco NM, Gjermo P, Preus HR. Clinical and microbiological effects of adjunctive antibiotics in treatment of localized juvenile periodontitis. A controlled clinical trial. *Journal of Periodontology* 1998; **69:** 1355-1363

Valenza G, Veihelmann S, Peplies J, Tichy D, Roldan-Pareja, Mdel C, Schlagenhauf U, Vogel U. Microbial changes in periodontitis successfully treated by mechanical plaque removal and systemic amoxicillin and metronidazole. *International Journal of Medical Microbiology* 2009; **299:** 427-438.

Van Winkelhoff AJ, Tijhof CJ, de Graaff J. Microbiological and clinical results of metronidazole plus amoxicillin therapy in *Actinobacillus actinomycetemcomitans-*associated periodontitis. *Journal of Periodontology* 1992; **63:** 52-57.

Winkel EG, Van Winkelhoff AJ, Van der Velden U. Additional clinical and microbiological effects of amoxicillin and metronidazole after initial periodontal therapy. *Journal of Clinical Periodontology* 1998; **25:** 857-864.
